# The *Caenorhabditis *globin gene family reveals extensive nematode-specific radiation and diversification

**DOI:** 10.1186/1471-2148-8-279

**Published:** 2008-10-09

**Authors:** David Hoogewijs, Sasha De Henau, Sylvia Dewilde, Luc Moens, Marjolein Couvreur, Gaetan Borgonie, Serge N Vinogradov, Scott W Roy, Jacques R Vanfleteren

**Affiliations:** 1 Department of Biology and Center for Molecular Phylogeny and Evolution, Ghent University, B-9000 Ghent, Belgium; 2 Department of Biomedical Sciences, University of Antwerp, B-2610 Antwerp, Belgium; 3 Department of Biology, Nematology section, Ghent University, 9000 Ghent, Belgium; 4 Department of Biochemistry and Molecular Biology, Wayne State University School of Medicine, Detroit, Michigan 48201, USA; 5 National Center for Biotechnology Information, National Library of Medicine, National Institutes of Health, Bethesda, MD 20814, USA

## Abstract

**Background:**

Globin isoforms with variant properties and functions have been found in the pseudocoel, body wall and cuticle of various nematode species and even in the eyespots of the insect-parasite *Mermis nigrescens*. In fact, much higher levels of complexity exist, as shown by recent whole genome analysis studies. *In silico *analysis of the genome of *Caenorhabditis elegans *revealed an unexpectedly high number of globin genes featuring a remarkable diversity in gene structure, amino acid sequence and expression profiles.

**Results:**

In the present study we have analyzed whole genomic data from *C. briggsae*, *C. remanei*, *Pristionchus pacificus *and *Brugia malayi *and EST data from several other nematode species to study the evolutionary history of the nematode globin gene family. We find a high level of conservation of the *C. elegans *globin complement, with even distantly related nematodes harboring orthologs to many *Caenorhabditis *globins. Bayesian phylogenetic analysis resolves all nematode globins into two distinct globin classes. Analysis of the globin intron-exon structures suggests extensive loss of ancestral introns and gain of new positions in deep nematode ancestors, and mainly loss in the *Caenorhabditis *lineage. We also show that the *Caenorhabditis *globin genes are expressed in distinct, mostly non-overlapping, sets of cells and that they are all under strong purifying selection.

**Conclusion:**

Our results enable reconstruction of the evolutionary history of the globin gene family in the nematode phylum. A duplication of an ancestral globin gene occurred before the divergence of the Platyhelminthes and the Nematoda and one of the duplicated genes radiated further in the nematode phylum before the split of the Spirurina and Rhabditina and was followed by further radiation in the lineage leading to *Caenorhabditis*. The resulting globin genes were subject to processes of subfunctionalization and diversification leading to cell-specific expression patterns. Strong purifying selection subsequently dampened further evolution and facilitated fixation of the duplicated genes in the genome.

## Background

Globins are small globular proteins, usually consisting of about 140–150 amino acids that comprise eight α-helical segments (named A-H), displaying a characteristic 3-over-3 α-helical sandwich structure that encloses an iron-containing heme group. Vertebrate globin genes predominantly contain three exons separated by two introns inserted at highly conserved positions B12.2 (intron located between codon positions 2 and 3 of the 12th amino acid of globin helix B) and G7.0 (intron inserted between the codons for amino acids 6 and 7 of helix G). Organisms can express multiple globin molecules that have variant properties and functions. Vertebrates typically express haemoglobin in red blood cells, myoglobin in muscle, neuroglobin in nervous tissue [1] and cytoglobin in a variety of non-neuronal cells [2]. Invertebrate globins constitute a more heterogeneous group in terms of structure and function. They range from single-domain globins to large, multisubunit, multidomain hemoglobins and can be fused with nonglobin subunits forming chimeric proteins. Besides a conventional O_2 _storage and transport function, a wealth of diverse functions has been described for invertebrate globins [3,4]. Nematodes express distinct globin isoforms in the pseudocoel, body wall and cuticle [5]. The emergence of whole genome analysis tools has revealed even higher levels of complexity. *In silico *analysis of the *C. elegans *genome identified 33 putative globin genes in this species using a robust alignment procedure based on conserved structural features of the classical globin fold. These globins feature a wide diversity in gene structure, amino acid sequence and expression profiles. Despite this remarkable variety some of them display significant sequence similarity to vertebrate myoglobin, neuroglobin and cytoglobin [6,7].

The availability of full genomic sequences of two additional *Caenorhabditis *species presents a unique opportunity to explore the evolutionary globin dynamics of these species. In this study we provide a comprehensive evolutionary analysis of the *Caenorhabditis *globin gene family and we document that globins are found in a broad range of other nematode species.

## Results and discussion

### Occurrence of globins in the Nematoda

We identified globin gene sequences and exon-structures from full genomic sequences of five nematodes: the *Caenorhabditis *species *C. elegans*, *C. briggsae *and *C. remanei *(Rhabditina, Rhabditidae) and the distantly related nematodes *Pristionchus pacificus *(Rhabditina, Diplogasteromorpha) and *Brugia malayi *(Spirurina, Spiruromorpha) (Additional file 1). This allows comparison of nematode globins across three levels – ortholog evolution within the *Caenorhabditis *genus, ortholog evolution across more distantly related nematodes and evolution of paralogous genes.

Ortholog conservation for *C. elegans *globins varied across levels. We identified clear orthologs of all 33 *C. elegans *genes in *C. briggsae *and *C. remanei*, but reciprocal blast searches with the TBLASTN algorithm [8] and the *C. elegans *globin amino acid sequences identified only 24 and 13 globins in the genomes of *Pristionchus pacificus *and *Brugia malayi*, respectively, reducing the number of orthologs shared by all 5 species to 10.

By screening NEMBASE [9] we found 103 parasitic globins in several other parasitic nematodes with E-values below e^-05^. Some matched sequences of already known nematode globins from GenBank, while others lacked parts of the A-or H-helix reducing the total number of different NEMBASE EST clusters to 85 (Table 1). Thus far, we can conclude that globins are present in 31 different nematode species representing 4 nematode clades (I, III, IV and V [10,11]). In addition to the species listed in Table 1, partial globin sequences were also detected in *Heterodera schachtii, Litomosoides sigmodontis, Meloidogyne chitwoodi *and *Strongyloides ratti*.

**Table 1:** Parasitic nematode EST clusters identified in NEMBASE

species	NEMBASE ID
*Ancylostoma caninum*	ACP00369_1
*Ancylostoma caninum*	ACP01487_2
*Ancylostoma caninum*	ACP03829_1
*Ascaris lumbricoides*	ALP00043_1
*Ascaris suum*	ASP00019_1
*Ascaris suum*	ASP00780_1
*Ascaris suum*	ASP17423_1
*Ancylostoma ceylanicum*	AYP00272_1
*Ancylostoma ceylanicum*	AYP00544_1
*Ancylostoma ceylanicum*	AYP02881_1
*Ancylostoma ceylanicum*	AYP01816_1
*Ancylostoma ceylanicum*	AYP03578_1
*Ancylostoma ceylanicum*	AYP03942_1
*Dirofilaria immiti*	DIP00455_1
*Dirofilaria immitis*	DIP00540_1
*Globodera rostochiensis*	GRP00115_1
*Globodera rostochiensis*	GRP00048_1
*Haemonchus contortus*	HCP00202_1
*Haemonchus contortus*	HCP00202_2
*Haemonchus contortus*	HCP00202_3
*Haemonchus contortus*	HCP00202_4
*Haemonchus contortus*	HCP00202_5
*Haemonchus contortus*	HCP00208_1
*Haemonchus contortus*	HCP00208_2
*Haemonchus contortus*	HCP00333_2
*Haemonchus contortus*	HCP00759_1
*Haemonchus contortus*	HCP00759_2
*Haemonchus contortus*	HCP00759_3
*Haemonchus contortus*	HCP00770_1
*Haemonchus contortus*	HCP00770_2
*Haemonchus contortus*	HCP00770_3
*Haemonchus contortus*	HCP00786_1
*Haemonchus contortus*	HCP01314_3
*Haemonchus contortus*	HCP02815_1
*Haemonchus contortus*	HCP08501_1
*Haemonchus contortus*	HCP13111_1
*Heterodera glycines*	HGP06223_2
*Heterodera glycines*	HGP00385_1
*Meloidogyne hapla*	MHP02564_2
*Meloidogyne hapla*	MHP04412_1
*Meloidogyne incognita*	MIP00586_2
*Meloidogyne javanica*	MJP04640_1
*Meloidogyne paranaensis*	MPP00429_1
*Necator americanus*	NAP00041_1
*Necator americanus*	NAP00088_1
*Nippostrongylus brasiliensis*	NBP00095_1
*Nippostrongylus brasiliensis*	NBP00124_1
*Nippostrongylus brasiliensis*	NBP00197_1
*Nippostrongylus brasiliensis*	NBP00328_1
*Ostertagia ostertagi*	OOP00190_1
*Ostertagia ostertagi*	OOP00190_2
*Ostertagia ostertagi*	OOP00214_1
*Ostertagia ostertagi*	OOP03092_2
*Ostertagia ostertagi*	OOP03348_2
*Ostertagia ostertagi*	OOP03513_1
*Onchocerca volvulus*	OVP00634_1
*Onchocerca volvulus*	OVP06929_1
*Onchocerca volvulus*	OVP04040_1
*Parastrongyloides trichosuri*	PTP03438_1
*Radophulus similis*	RSP00034_1
*Strongyloides stercoralis*	SSP00231_1
*Strongyloides stercoralis*	SSP00309_1
*Strongyloides stercoralis*	SSP02226_1
*Strongyloides stercoralis*	SSP04285_1
*Strongyloides stercoralis*	SSP04654_1
*Toxocara canis*	TCP00537_1
*Teladorsagia circumcincta*	TDP00008_1
*Teladorsagia circumcincta*	TDP00008_2
*Teladorsagia circumcincta*	TDP00008_3
*Teladorsagia circumcincta*	TDP00008_4
*Teladorsagia circumcincta*	TDP00009_1
*Teladorsagia circumcincta*	TDP00009_2
*Teladorsagia circumcincta*	TDP00032_1
*Teladorsagia circumcincta*	TDP00084_1
*Teladorsagia circumcincta*	TDP00127_1
*Teladorsagia circumcincta*	TDP00173_1
*Teladorsagia circumcincta*	TDP01113_1
*Teladorsagia circumcincta*	TDP01113_2
*Trichuris muris*	TMP00180_1
*Trichuris muris*	TMP01615_1
*Trichinella spiralis*	TSP03467_1
*Trichuris vulpis*	TVP00077_1
*Trichuris vulpis*	TVP00688_1
*Xiphinema index*	XIP00721_1
*Zeldia punctata*	ZPP00218_1

Accession numbers correspond to those in NEMBASE.

### Two separate globin classes?

**Figure 1:**
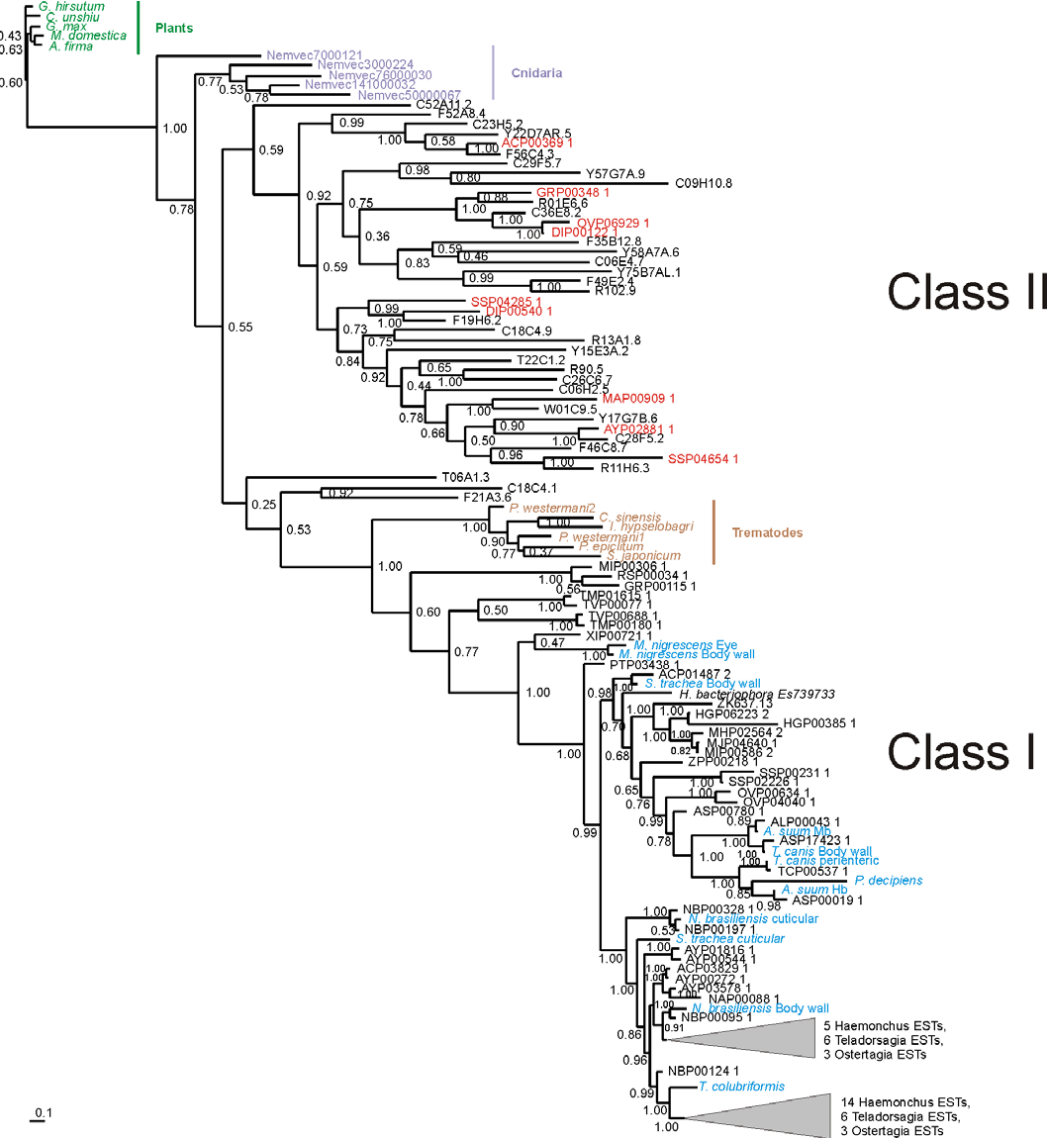
**Bayesian phylogenetic tree of globins from *C. elegans *and from 30 other nematode, predominantly parasitic, species.** Orthologs from parasitic species in clade II are marked in red. Clade I globins from parasitic nematodes that have been known for long because of their high abundance are marked in blue. The numbers at the nodes represent Bayesian posterior probabilities. All accession numbers from NEMBASE start with the initials of the species followed by P: *Ancylostoma caninum (AC), Ancylostoma ceylanicum (AY), Ascaris lumbricoides (AL), Ascaris suum (AS), Dirofilaria immitis (DI), Globodera rostochiensis (GR), Haemonchus contortus (HC), Heterodera glycines (HG), Meloidogyne chitwoodi (MC), Meloidogyne hapla (MH), Meloidogyne incognita (MI), Meloidogyne javanica (MJ), Necator americanus (NA), Nippostrongylus brasiliensis (NB), Ostertagia ostertagi (OO), Onchocerca volvulus (OV), Parastrongyloides trichosuri (PT), Strongyloides stercoralis (SS), Toxocara canis (TC), Teladorsagia circumcincta (TD), Trichuris muris (TM), Trichinella spiralis (TS), Trichuris vulpis (TV), Xiphinema index (XI), Zeldia punctata (ZP)*. Plant, trematode (Platyhelminthes) and sea anemone (Cnidaria) globins were included as outgroup globins. The following plant globins (green) were included:*Gossypium hirsutum (*AAX86687), *Malus domestica *(AAP57676), *Glycine max *(AAA97887), *Alnus firma *(BAE75956), *Citrus unshiu *(AAK07675). The trematode globins (brown) were: *Schistosoma japonicum *(AAP06216), *Paramphistomum epiclitum *(AAG48877), *Paragonimus westermani *(AAX11352 and AAX11353), *Clonorchis sinensis *(AAM18464), *Isoparorchis hypselobagri *(P80722). *Nematostella vectensis *globins (purple): Nemvec141000032, Nemvec3000224, Nemvec50000067, Nemvec7000121 and Nemvec76000030.

Overall, we recovered over 120 new globin-like sequences from exhaustive database searches. Globin domains were extracted, aligned (Additional files 2 and 3) and subjected to phylogenetic analysis. Bayesian inference of the globin domains of all 33 *C. elegans *globins and globins from a representative set of 26 non-Rhabditid, mostly parasitic, species and globins from plants, trematodes and a sea anemone (*Nematostella vectensis*) as outgroup taxa clearly separated two classes of nematode globins. Class I globins comprises *C. elegans *ZK637.13, well known globins from other nematode species and novel related nematode globin isoforms as well as the trematode globins and is supported by 100% posterior probability. Class II globin genes consist of the remaining 32 *Caenorhabditis *globins and novel orthologs thereof, identified in parasitic nematodes (Figure 1). They form a more diverse clade that is resolved from the sea anemone globins with moderate support (p = 0.78). This pattern was essentially retained, though not satisfactorily supported, when more globins from distantly related eukaryotes were included in the analysis (results not shown). We included the cnidarian and trematode globins in this analysis because a recent phylogenomic analysis resolved the Cnidaria as a sister taxon to the Bilateria and the Lophotrochozoa (comprising the Platyhelminthes) as a sister taxon to the Ecdysoza, to which the Nematoda belong [12]. Thus Bayesian inference seems to indicate that a class II ancestral globin evolved before the divergence of the Platyhelminthes and the Nematoda and radiated further in the nematode phylum. Alternative phylogenetic methods, neighbor-joining (MEGA [13]) and maximum likelihood (RAxML [14]), were consistent with Bayesian analysis but statistical support was not significant (data not shown).

**Figure 2:**
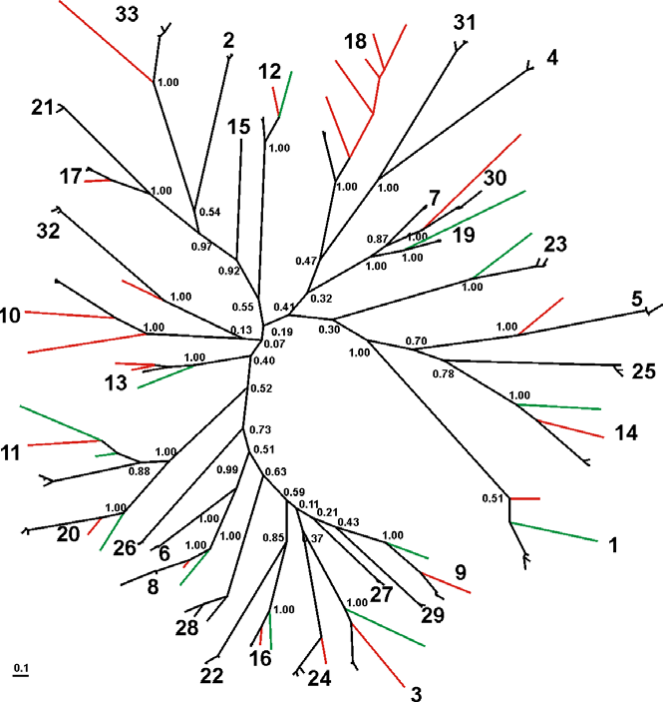
**Unrooted bayesian phylogenetic tree of all *C. elegans, C. briggsae, C. remanei, Brugia malayi *(labeled in green) and *Pristionchus pacificus *(labeled in red) globins.** The numbers at the nodes represent Bayesian posterior probabilities. The numbers at the branches are consistent with globin nomenclature in WormBase: ZK637.13 (1), C06E4.7 (2), C06H2.5 (3), C09H10.8 (4), C18C4.1 (5), C18C4.9 (6), C23H5.2 (7), C26C6.7 (8), C28F5.2 (9), C29F5.7 (10), C36E8.2 (11), C52A11.2 (12), F19H6.2 (13), F21A3.6 (14), F35B12.8 (15), F46C8.7 (16), F49E2.4 (17), F52A8.4 (18), F56C4.3 (19), R01E6.6 (20), R102.9 (21), R11H6.3 (22), R13A1.8 (23), R90.5 (24), T06A1.3 (25), T22C1.2 (26), W01C9.5 (27), Y15E3A.2 (28), Y17G7B.6 (29), Y22D7AR.5 (30), Y57G7A.9 (31), Y58A7A.6 (32), Y75B7AL.1 (33).

**Figure 3:**
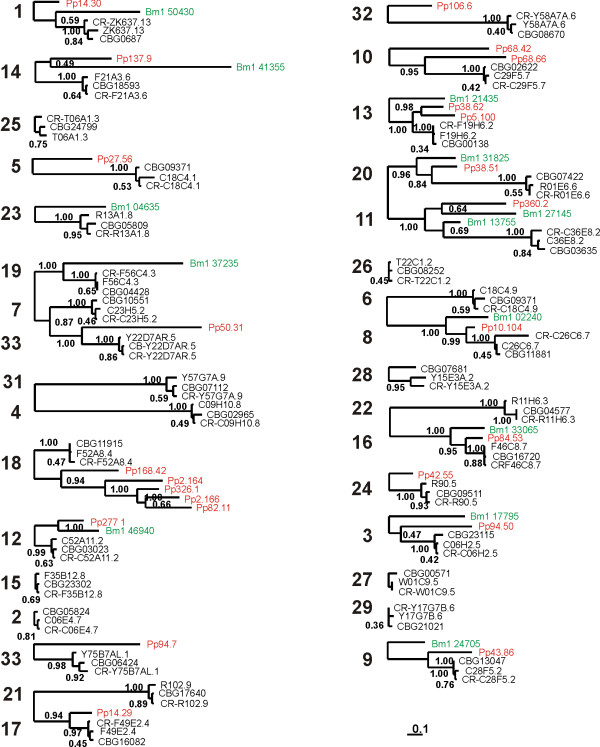
**Detailed view of Figure 2, phylogenetic relationship of all orthologous groups of *C. elegans, C. briggsae, C. remanei, Brugia malayi *(labeled in green) and *Pristionchus pacificus *(labeled in red) globins.** Due to the complex WashU nomenclature ID, *C. remanei *globins are referred to as *C. elegans *homologue preceded by 'CR-'. The numbers at the nodes represent Bayesian posterior probabilities.

The finding that 9 parasite EST's resolve with class II globins feeds our expectation that many more are to be detected as more nematode genome sequences will become available. The phylogenetic trees represented in Figure 2 and Figure 3 clearly illustrate that the divergence of the globin genes preceded species divergence. Also, molecular and species trees (*Brugia *separates first, followed by *Pristionchus *and next the *Caenorhabdits *cluster) agree very well for most globin genes. Of note are the very short branch lengths from the *Caenorhabditis *ancestor to the three extant species, considering that these species diverged around 100 million year ago [15] (Figure 3). These branch lengths are disproportionally short, relative to the branch lengths generated for *Brugia *and *Pristionchus *and even more so to those representing the divergence of the globin genes. A likely explanation is that the rate of evolution of the globin genes drastically slowed down in *Caenorhabditis*. Alternatively, diversity within *Caenorhabditis *could represent less of nematode history (i.e. a much more recent divergence) than previously appreciated. To distinguish between these possibilities we explored the rate of evolution of several other gene families including astacins, superoxide dismutases, glutathione S-transferases, nicotinic acetylcholine receptors, hedgehog-related proteins and ATP-binding proteins. We assembled a matrix of as many as possible five-way orthologs for the five taxa and analyzed the resulting matrix by Bayesian inference using partitioning of the data (Additional file 4). The ratios of the branch lengths from *Brugia malayi *or *Pristionchus pacificus *to the *Caenorhabditis *internal node and the average branch length of that node to the extant species suggest that the evolutionary rate is quite variable among gene families (Table 2). However, these results also suggest that four (globins, nicotinic acetylcholine receptors, astacins and hedgehog-related proteins) out of the seven multigene families tested evolved more slowly in the *Caenorhabditis *lineage.

**Table 2:** Evolutionary distance of seven multigene families of *Pristionchus pacificus *and *Brugia malayi *compared to *Caenorhabditis*, inferred from branch length ratios (Additional file 4). *glb *globin; *gst *glutathione S-transferase; *ast *astacin, *sod *superoxide dismutase, *acr *nicotinic acetylcholine receptors; *wrt *hedgehog-related (warthog) proteins; *abc *ATP-binding proteins.

	*Glb*	*gst*	*sod*	*ast*	*wrt*	*acr*	*abc*
***B. malayi***	14.10526	7.09375	7.439024	12.15217	10.34615	17.60357	4.163636
***P. pacificus***	10.86842	5.59375	4.182927	10.65217	11.15385	16.96071	4.272727

### Tissue-specific expression

**Figure 4:**
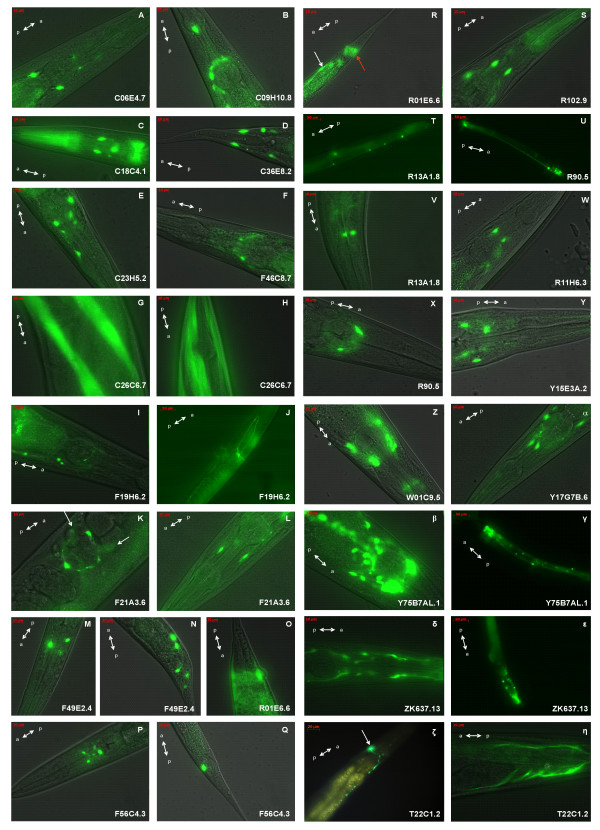
**Expression patterns from a selected set of globin genes.** A, anterior body part; P, posterior body part. (**A**) C06E4.7, (**B**) C09H10.8, (**C**) C18C4.1, (**D**) C36E8.2, (**E**) C23H5.2, (**F**) F46C8.7, (**G**) C26C6.7, (**H**) C26C6.7, (**I**) F19H6.2, (**J**) F19H6.2, (**K**) F21A3.6 white arrows denote vulval muscle, (**L**) F21A3.6, (**M**) F49E2.4, (**N**) F49E2.4, (**O**) R01E6.6, (**P**) F56C4.3, (**Q**) F56C4.3, (**R**) R01E6.6, white arrow denotes stomato-intestinal muscle, red arrow denotes anal depressor muscle, (**S**) R102.9, (**T**) R13A1.8, (**U**) R90.5, (**V**) R13A1.8, (**W**) R11H6.3, (**X**) R90.5, (**Y**) Y15E3A.2, (**Z**) W01C9.5, (**α**) Y17G7B.6, (**β**) Y75B7AL.1, (**γ**) Y75B7AL.1, (**δ**) ZK637.13, (**ε**) ZK637.13, (**ζ**) T22C1.2, white arrow denotes head mesodermal cell, (**η**) T22C1.2, stomato-intestinal muscle.

We constructed gene fusions of approx 800 – 3000 bp upstream promoter and enhancer regions for all 33 globin genes to the coding region of GFP. Spatial expression patterns were observed for 32 genes. Although we have not yet unambiguously identified the specific cells expressing these genes it is clear that these globins are expressed in distinct, mostly non-overlapping sets of cells. Most globin genes were expressed in neuronal cells in the head and tail portions of the body, and the nerve cord. A minority of the globin genes were expressed in non-neuronal tissues, including body wall and vulval muscle and the pharynx; only one globin gene, C26C6.7 was expressed in muscular tissue exclusively. We never observed GFP expression in the intestine or gonad (Table 3 and Figure 4).

**Table 3:** Overview of globin expression patterns

Gene	Expression pattern
C06E4.7	Head neurons
C06H2.5	Head and tail neurons, nerve cord
C09H10.8	Head and tail neurons, nerve cord
C18C4.1	Pharynx (Corpus, isthmus), head neurons, nerve cord
C18C4.9	Head and tail neurons, nerve cord
C23H5.2	Head and tail neurons
C26C6.7	Body wall muscle
C28F5.2	Head and tail neurons, nerve cord, vulva neurons
C29F5.7	Head and tail neurons, nerve cord
C36E8.2	Pharynx (Corpus, terminal bulb), head and tail neurons, nerve cord
C52A11.2	Head and tail neurons
F19H6.2	Head neurons, nerve cord, vulva neuron
F21A3.6	Head neurons, vulva neurons, vulval muscle
F35B12.8	No observable expression
F46C8.7	Head neurons, tail neuron
F49E2.4	Head and tail neurons, nerve cord
F52A8.4	Head and tail neurons, nerve cord
F56C4.3	Head neurons and tail neuron
R01E6.6	Stomato-intestinal muscle, depressor muscle, body wall muscle, head neurons, vulva neurons, nerve cord
R102.9	Pharynx (Corpus), head neurons, nerve cord
R11H6.3	Head and tail neurons, nerve cord
R13A1.8	Head neurons, nerve cord
R90.5	Head and tail neurons, nerve cord
T06A1.3	Head and tail neurons, nerve cord
T22C1.2	Head mesodermal cell, stomato-intestinal muscle
W01C9.5	Head and tail neurons, nerve cord
Y15E3A.2	Head and tail neurons, nerve cord
Y17G7B.6	Head and tail neurons, nerve cord
Y22D7AR.5	Head and tail neurons, nerve cord
Y57G7A.9	Head neurons
Y58A7A.6	Head and tail neurons, nerve cord
Y75B7AL.1	Head and tail neurons, nerve cord
ZK637.13	Head muscle/hypodermis, tail muscle/hypodermis, head, tail and vulva neurons, Nerve cord

### Intron evolution

**Figure 5:**
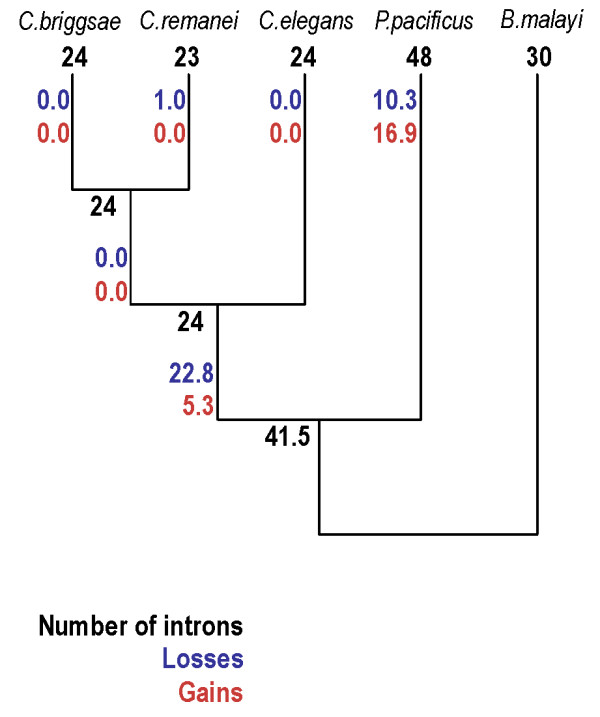
Estimates of intron losses and gains.

Analysis of the gene structures for conserved globin domains for the 10 five-way conserved orthologs revealed striking differences across evolutionary levels (Table 4). Consistent with previous findings (e.g. [16]), most intron positions (23/24) were conserved across *Caenorhabditis *species. Orthologous gene structures were more diverged between genera, but majorities of positions were shared across genera for each species (17/24 *Caenorhabditis *positions were also identified in *Pristionchus *and/or *Brugia*, 25/48 *Pristionchus pacificus *insertion positions in *Caenorhabditis *and/or *Brugia*, and 23/30 *B. malayi *positions in *Caenorhabditis *and/or *Pristionchus*). To determine the relative contribution of intron loss and gain to these patterns, we used previously published methods [17,18] to reconstruct evolution (Figure 5). Estimated numbers of gains and losses were similar (22 versus 34), in stark contrast to patterns observed within *Caenorhabditis *[19-22]. These reconstructions suggest very different histories in *Caenorhabditis *(four losses for each gain) and *P. pacificus *(60% more gains than losses).

**Table 4:** Overview of intron insertion positions in the globin domain for all five-way conserved orthologs

Globin	*C. elegans*	*C. briggsae*	*C. remanei*	*B. malayi*	*P. pacificus*
***C06H2.5***	A2.2 G15.0	A2.2 G15.0	A2.2 G15.0	A2.2 B11.2 EF11.0 H16.0	A15.0 B11.0 E9.1 EF11.0 G15.0

***C26C6.7***	C4.0 GH4.2	C4.0 GH4.2	C4.0 GH4.2	E18.0 FG1.0	B1.0 C4.0 E18.0 G2.1 GH4.2

***C28F5.2***	E13.2 FG5.0	E13.2 FG5.0	E13.2 FG5.0	C7.0 E13.2 FG5.0	AB9.1 B1.0 C7.0 E13.2 F8.2 H1.0

***C36E8.2***	E6.0 EF14.2 H22.2	E6.0 EF14.2 H22.2	EF14.2 H22.2	E2.0 H8.2	E2.0 EF11.0 H21.1

***C52A11.2***	E18.0 FG2.2 GH2.0	E18.0 FG2.2 GH2.0	E18.0 FG2.2 GH2.0	A14.0 C7.0 E18.0 GH6.0	A14.0 C7.0 E18.0 F7.0 GH6.0 H10.2

***F19H6.2***	AB2.0 E10.1 H12.0	AB2.0 E10.1 H12.0	AB2.0 E10.1 H12.0	AB2.0 F3.0 H12.0	A3.2 D6.2 F3.0 G15.0 H12.0

***F21A3.6***	B12.2 E5.2 G7.0	B12.2 E5.2 G7.0	B12.2 E5.2 G7.0	B12.2 G7.0	B12.2 E17.0 G7.0

***F46C8.7***	B9.0 F5.1	B9.0 F5.1	B9.0 F5.1	B9.0 E10.0 F5.1 H19.0	A7.0 B9.0 CD4.2 E10.0 F5.1 GH2.1

***R01E6.6***	B9.0 E16.0 H1.0	B9.0 E16.0 H1.0	B9.0 E16.0 H1.0	C6.2 E16.0 H1.0	B9.0 E16.0 FG2.1 H1.0

***ZK637.13***	E3.2	E3.2	E3.2	B12.2 EF2.1 G7.0	B12.2 E3.2 EF7.0 G7.0 H15.1

The most striking pattern is seen at the level of divergence between paralogs. In contrast to the generally conserved intron positions of most eukaryotic globins, nematode globins exhibit a tremendous variability in intron positions (Additional file 1). Interestingly, only one *Caenorhabditis *globin (F21A3.6) displays the typical ancestral intron positions common to vertebrates and other eukaryotes (at positions B12.2 and G7.0), as do the *B. malayi *and *P. pacificus *orthologs. In addition, the *Brugia *ortholog of *C. elegans *globin gene ZK637.13, which has only one intron inserted at E3.2, has B12.2 and G7.0, whereas the *Pristionchus *ortholog contains B12.2 and G7.0 as well as E3.2. Finally, we found no strong evidence for or against any particular model of intron gain – consistent with the apparent age of the introns (most dating to early nematodes), no clear sequence similarity was observed to other genomic sequences (as expected by transposition of existing introns or intron creation by transposable element insertion) or to flanking exonic sequences (as expected by transformation of duplicated genomic sequence into a new intron). The diversity of globin intron positions in nematodes stands in stark contrast to that observed in some other lineages. For instance, in vertebrates, despite a degree of sequence divergence among paralogous globins comparable to that in nematodes, intron positions are generally conserved. Notably, intron-exon structure of nematode globins follows protein divergence; F21A3.6 is both the only gene to retain both ancestral introns, and shows the highest sequence identity to vertebrate globins (specifically, vertebrate myoglobin; data not shown).

In total, then, nematode globin intron-exon structures suggest a large amount of intron change (loss of ancestral introns and gain of new positions) in deep nematode ancestors, followed by differential loss and gain in individual subsequent lineages, with the *Caenorhabditis *lineage showing a pronounced excess of loss over gain. These data deepen the mystery of atypical nematode intron evolution (including high rates of loss and gain, frequent trans-splicing, and atypical intron splicing signals).

### Mode of selective pressure

The striking diversity of nematode globins raises the question of the role of natural selection on the evolution of these genes. We determined the ratio of non-synonymous (Ka) to synonymous substitution (Ks) rates for each orthologous *briggsae/elegans *pair (Table 5). The substitution ratio (ω) is expected to be near 1 for genes under neutral selection, greater than 1 for genes under positive selection and smaller than 1 for those under negative selection. All ω-ratios ranged from 0.0070 to 0.1876 with an average of 0.0367, indicating functional constraint. Similar results were found for the orthologous *elegans/remanei *(average of 0.0359) and *briggsae/remanei *(0.0463 on average) pairs. No substantial differences were obtained when this analysis was applied on the globin domains only (results not shown). All *C. elegans *globin genes are expressed [7]. Although pseudogenes have been found in *C. elegans *with ω-ratios down to 0.4 (Ian Hope, personal communication), the combination of these findings refutes the possibility that some of them would be pseudogenes.

**Table 5:** Overview of K_A_/K_S _(ω)-ratios for *C. briggsae – C. elegans *orthologs

globin	K_A_**/K**_S_
*C06E4.7*	0.0171
*C06H2.5*	0.0184
*C09H10.8*	0.0115
*C18C4.1*	0.0248
*C18C4.9*	0.0295
*C23H5.2*	0.0376
*C26C6.7*	0.0223
*C28F5.2*	0.0199
*C29F5.7*	0.0172
*C36E8.2*	0.0519
*C52A11.2*	0.0400
*F19H6.2*	0.0096
*F21A3.6*	0.0535
*F35B12.8*	0.0070
*F46C8.7*	0.0260
*F49E2.4*	0.0178
*F52A8.4*	0.0318
*F56C4.3*	0.0274
*R01E6.6*	0.0473
*R102.9*	0.0344
*R11H6.3*	0.0251
*R13A1.8*	0.0372
*R90.5*	0.0292
*T06A1.3*	0.0381
*T22C1.2*	0.0506
*W01C9.5*	0.0153
*Y15E3A.2*	0.1876
*Y17G7B.6*	0.0173
*Y22D7AR.5*	0.0287
*Y57G7A.9*	0.0274
*Y58A7A.6*	0.0093
*Y75B7AL.1*	0.1102
*ZK637.13*	0.0849

We also asked whether the large N- and C-terminal extensions of many *C. elegans *globins are required for their specific function. To this end we searched for positive selection on individual amino acids, because positive selection is thought to act only on specific residues in a protein that is under purifying selection [23]. No positive selection was detected using the maximum likelihood procedure of Thomas et al. [24,25], even in globin Y75B7AL.1, which is a chimeric polypeptide composed of a C-terminal globin domain and an N-terminal domain that has all characteristics of a G-coupled sensor. This domain contains 7 transmembrane helices and this structure is a candidate target for positive selection [25]. The strong purifying selection acting on the globin genes is consistent with the slowing of the rate of evolution of the exon-structures in the genus *Caenorhabditis*.

## Conclusion

Our study unveils an unexpected complexity of the globin family in nematodes. *Caenorhabditis *species contain a very large number of globin genes, and even distantly related nematode species harbor orthologs to many of them. Our analysis provides some evidence for a number of gene duplication events giving rise to a class of globin genes that is likely unique to the nematode phylum. It remains to be seen whether this class will persist as more genomes will become available.

It is generally accepted that gene duplication played a major role in the evolution of eukaryotic genomes, particularly in the origin of multi-gene families [26]. Duplication events that gave rise to the radiation of nematode globin genes most likely occurred too long ago to leave behind obvious evidence of adaptive evolution. The dispersion of the globin genes over all six chromosomes of *C. elegans *is consistent with this idea. Globins R102.9 and F49E2.4 are exceptional in showing strong sequence conservation (Figure 2) and sharing 2 intron positions (Additional file 1). However, they are located on different chromosomes, arguing against a very recent tandem duplication event. Similarly, both *C. briggsae *orthologs are found on different chromosomes.

Why would tiny animals like *Caenorhabditis *species need up to 33 different globins? We have shown that they are all expressed and subject to strong purifying selection. Extensive gene duplication appears to be typical for *Caenorhabditis*. Other surprisingly large gene families include astacins [27], insulins [28], chemoreceptors [20,21] and orphan nuclear receptors HNF4 [29]. Subfunctionalization is frequently invoked to explain the retention of duplicate genes, and differences in gene expression patterns of duplicate genes are generally advanced in support of this hypothesis [30-33]. It is likely that this mechanism was the driving force for the expansion of the *Caenorhabditis *globin family. We have demonstrated that these genes are expressed in distinct subsets of cells and that they are subject to strong purifying selection, in line with this hypothesis. Further support is provided by differential expression of subsets of globin genes in the dauer stage and upon oxygen deprivation [7]. Evidence is mounting that behavioral responses of *C. elegans *to attracting or repelling chemicals including oxygen and CO_2 _are generated by gene activities that are deployed in different combinations of neurons [34-37]. Individual worm neurons seem to have attained very high specialization which is in keeping with their expressing unique or small subsets of globin isoforms.

To date the precise function of none of these globins is known. It seems unlikely that they would all be required for simple oxygen transportation or storage purposes. Globins that are upregulated when oxygen supply is low might serve this function [7]. Other functions are also plausible. It has been shown that *C. elegans *senses molecular oxygen through the heme domain of a guanylate cyclase homolog (GCY-35) and reacts rapidly to changing oxygen levels with aerotaxis responses [37,38]. We expect that some globins may play similar roles in distinct sensory neurons. One likely candidate is globin Y75B7AL.1 which has all characteristics of a G-coupled sensor. Still other functions are worth consideration. Globin T22C1.2 oxidizes instantly to the ferric form in the presence of oxygen and is therefore not capable of reversible oxygen binding (unpublished results). We speculate that this globin may participate in redox reactions with an as yet unidentified reaction partner. Alternatively, peroxidase activity which is a latent but inherent property of globins might have evolved to become the ultimate function of other *C. elegans *globins. Finally, we cannot exclude a potential role as an alternative oxidase during anaerobiosis, when levels of oxygen drop below saturation of cytochrome oxidase, as has been well documented for plant cells [39,40]. Future research shall provide more answers.

## Methods

### Sequence database searches

The BLAST algorithm [8] was employed to search the sequence databases WormBase (Release WS182), TIGR (*Brugia malayi*) and http://www.pristionchus.org (Assembly Freeze 1). NEMBASE 3, containing EST clusters from 37 different partial parasitic nematode genomes [9], was searched for globin motifs (PF00041, PS01033 and SSF46458). Additional EST sequences were searched from EMBL-EBI parasite EST, and the NCBI parasite EST databases using the BLAST algorithm [8] with the *Caenorhabditis *globins as query sequences and cut off E-values of e^-05^. In cases where the identification of a putative globin was uncertain, searches employing FUGUE [41] were used to determine whether the borderline sequence should be accepted as a globin.

Additional sequences of known nematode globins were obtained from GenBank: *Trichostrongylus colubriformis *(AAA30102), *Nippostrongylus brasiliensis *cuticular globin (P51536), *Nippostrongylus brasiliensis *body wall globin (P51535),*Toxocara canis *body wall globin (AAL56428), *Toxocara canis *perienteric globin (AAL56430), *Ascaris suum *myoglobin (AAA64695), *Ascaris suum *hemoglobin (AAA29374), *Mermis nigrescens *eye globin (AAF34874),*Mermis nigrescens *body wall globin (AAF35435), *Syngamus trachea *cuticular globin (AAL56426), *Syngamus trachea *body wall globin (AAL56427), *Pseudoterranova decipiens *(P26914). All encoded globin sequences were aligned manually as described previously [42,43].

### Globin gene expression analysis

The putative promoter and enhancer sequences upstream of the predicted start codon of each globin gene were extracted from the UCSC Genome Browser database using a repeat masker function [44]. PCR primers were designed to amplify about 3 kb of N2 wild-type genomic DNA, this size was reduced as needed to exclude any upstream gene located at a shorter distance. Promotor-GFP fusion constructs were generated as described by [45]. pRF4 *rol-6(su1006) *plasmid and promoter-reporter fusion construct were co-injected at 100 ng/μl and 50 ng/μl, respectively, into the gonads of young adult hermaphrodites. Rolling F1 worms were transferred to fresh plates and rolling F2 progeny were examined for fluorescence using an Axiovert 200 M (Zeiss) fluorescence microscope. At least 2 independent transgenic lines were examined for each globin gene. These lines generally displayed very similar GFP expression patterns. Primer sequences are available upon request.

### Pairwise estimates of K_A _and K_S_

After manual alignment of orthologous *briggsae/elegans *globin pairs, alignments of corresponding coding sequences were used to calculate the ratios (ω) of non-synonymous (*K*_A_) to synonymous substitutions (*K*_S_). We used the Yang and Nielsen [46] maximum likelihood method implemented in the YN00 program of the PAML package version 3.15 [47] since the Nei and Gojobori [48] method was not applicable in some cases. Similar results were generated in cases where both methods were applicable.

### Phylogenetic analysis

Bayesian inference trees were obtained employing MrBayes version 3.1.2 [49]; four chains were run simultaneously for 4 × 10^6 ^generations and trees were sampled every 100 generations generating a total of 40000 trees. The final average standard deviations of split frequencies were stationary and in the range of about 0.016. Posterior probabilities were estimated on the final 30000 trees. The appropriate model of amino acid sequence evolution (JTT model [50]) was selected by ProtTest [51] using the Akaike Information Criterion (AIC).

## Authors' contributions

DH and JRV conceived and designed the study; DH and SDH carried out data collection and genomic analysis and generated fusion constructs; DH and MC performed micro-injection experiments; DH and SWR performed data analysis; SD, LM, GB and SNV provided additional input in data analysis; DH, SWR and JRV wrote the manuscript. All authors read and approved the final manuscript.

## Supplementary Material

Additional file 1 **Overview of intron insertion positions in *Caenorhabditis*, *Brugia malayi *and *Pristionchus pacificus *globins. **Identical *Caenorhabditis*-*Pristionchus *intron positions are marked in red, identical *Brugia-Pristionchus *intron postions in blue and identical *Brugia-Caenorhabditis *intron postions in green. Phase 0 introns separate two consecutive codons (annotated by the number of the amino acid residue, a dot and number 0). Phase 1 and phase 2 introns are inserted following the first or second base of a codon, respectively (annotated by the number of the amino acid, a dot and number 1 or 2, respectively). Introns inserted in the N-terminal extensions are referred to as NA counting from amino acid 1, HC for those inserted in the C-terminal extension starting with the first amino acid after the H-helix. Click here for file

Additional file 2 Manual alignment of nematode globins. Click here for file

Additional file 3 **Similarity matrix of all 33 *C****. elegans *globins based on the alignment provided in Additional file 2. Click here for file

Additional file 4 Unrooted bayesian trees based on matrices of five-way orthologs from *C. elegans*, *C. briggsae, C. remanei, Pristionchus pacificus *and *Brugia malayi. glb *globin; *gst *glutathione S-transferase; *ast *astacin, *sod *superoxide dismutase, *acr *nicotinic acetylcholine receptors; *wrt *hedgehog-related (warthog) proteins; *abc *ATP-binding proteins. Click here for file
