# Supplementary material for: The Caenorhabditis globin gene family reveals extensive nematode-specific radiation and diversification
Source: BMC Evol Biol. 2008 Oct 9;8:279. doi: 10.1186/1471-2148-8-279 (PMC2576238; doi:10.1186/1471-2148-8-279)
Supplement: Additional file 1 — Overview of intron insertion positions in Caenorhabditis, Brugia malayi and Pristionchus pacificus globins. Identical Caenorhabditis-Pristionchus intron positions are marked in red, identical Brugia-Pristionchus intron postions in blue and identical Brugia-Caenorhabditis intron postions in green. Phase 0 introns separate two consecutive codons (annotated by the number of the amino acid residue, a dot and number 0). Phase 1 and phase 2 introns are inserted following the first or second base of a codon, respectively (annotated by the number of the amino acid, a dot and number 1 or 2, respectively). Introns inserted in the N-terminal extensions are referred to as NA counting from amino acid 1, HC for those inserted in the C-terminal extension starting with the first amino acid after the H-helix. [file 1471-2148-8-279-S1.doc]

**Additional file 1**

| *C. elegans* | | | *C. briggsae* | | *C. remanei* | | *Brugia malayi* | | | *Pristionchus pacificus* | | |
| --- | --- | --- | --- | --- | --- | --- | --- | --- | --- | --- | --- | --- |
| Gene name | Intron nr | Insertion positions | Intron nr | Insertion positions | Intron nr | Insertion positions | Gene name | Intron nr | Insertion positions | Gene name | Intron nr | Insertion positions |
| ***C06E4.7*** | **5** | A14.2 CD3.1 E18.0 GH7.1 H26.0 | **5** | A14.2 CD3.1 E18.0 GH7.1 H26.0 | **5** | A14.2 CD3.1 E18.0 GH7.1 H26.0 | / |  |  | / |  |  |
| ***C06H2.5*** | **2** | A2.2 G15.0 | **2** | A2.2 G15.0 | **2** | A2.2 G15.0 | Bm1_17795 | 5 | NA2.2 A2.2 B11.2 EF11.0 H16.0 | contig94.50 | 7 | NA36.2 NA53.1 A15.0 B11.0 E9.1 EF11.0 G15.0 |
| ***C09H10.8*** | **6** | A9.2 B11.2 F7.2 GH6.2 HC13.2 HC42.1 HC93.2 | **6** | A9.2 B11.2 F7.2 GH6.2 HC13.2 HC42.1 HC93.2 | **6** | A9.2 B11.2 F7.2 GH6.2 HC13.2 HC42.1 HC93.2 | / |  |  | / |  |  |
| ***C18C4.1*** | **9*** | NA20.0 NA43.1 NA67.0 B3.0 E6.2 H11.0 | **8** | NA34.0 NA57.1 NA81.0 B3.0 E6.2 H11.0 HC65.1 HC110.2 | **8** | NA21.0 NA48.1 NA68.0 B3.0 E6.2 H11.0 HC78.1 HC123.2 | / |  |  | Contig27.56 | 8 | NA28.0 B3.0 E6.2 G6.0 H11.0  HC20.0 HC43.0 HC78.0 |
| ***C18C4.9*** | **8** | NA44.1 NA72.1 NA106.1 NA 137.2 NA185.1 FG6.1 H3.0 HC12.0 | **7** | NA44,1 NA138,2 NA186,1 E6,1 FG6,1 H3,0 HC12,0 | **9** | NA44,1 NA72,1 NA106,1 NA137,2 NA185,1 E6,0 FG6,1 H3,0 HC12,0 | / |  |  | / |  |  |
| ***C23H5.2*** | **5** | C7.0 E14.0 F6.2 GH4.0 HC76.1 | **5** | C7.0 E14.0 F6.2 GH4.0 HC76.1 | **5** | C7.0 E14.0 F6.2 GH4.0 HC76.1 | / |  |  | / |  |  |
| ***C26C6.7*** | **7** | NA4.1 NA76.2 NA106.0 NA187.0 C4.0 GH4.2 HC1.0 | **7** | NA75.2 NA83.2 NA105.0 NA191.0 C4.0 GH4.2 HC1.0 | **6** | NA36.1 NA109.2 NA130.0 C4.0 GH4.2 HC1.0 | BM1_02240 | 6 | NA8.2 NA40.0 NA126.0 E18.0 FG1.0 HC2.0 | contig10.104 | 6 | B1.0 C4.0 E18.0 G2.1 GH4.2 HC1.0 |
| ***C28F5.2*** | **5** | NA23.1 NA45.1 E13.2 FG5.0 HC5.2 | **5** | NA23.1 NA45.1 E13.2 FG5.0 HC5.2 | **5** | NA23.1 NA45.1 E13.2 FG5.0 HC5.2 | Bm1_24705 | 5 | NA19.1 C7.0 E13.2 FG5.0 HC5.2 | Contig43.86 | 9 | NA17.2 AB9.1 B1.0 C7.0 E13.2 F8.2 H1.0 HC1.0 HC33.0 |
| ***C29F5.7*** | **3** | A14.2 E14.0 H10.2 | **3** | A14.2 E14.0 H10.2 | **3** | A14.2 E14.0 H10.2 | / |  |  | contig68.42 | 8 | NA35.1 NA64.2 B7.0 E3.0 F10.1 G10.0 H10.2 CH15.1 |
|  |  |  |  |  |  |  | / |  |  | contig68.66 | 7 | A14.2 C6.0 E19.0 F3.0 G10.0 H8.0 CH11.0 |
| ***C36E8.2*** | **4** | .NA26.2 E6.0 EF14.2 H22.2 | **4** | NA26.2 E6.0 EF14.2 H22.2 | **4** | NA26.2 EF14.2 H22.2 HC36.2 | Bm1_13755 | 3 | E2.0 H8.2 HC36.2 | contig360.2 | 7 | NA42.2 E2.0 EF11.0 H21.1 HC14.2 HC32.2 HC40.1 |
|  |  |  |  |  |  |  | Bm1_27145 | 3 | EF8.0 H1.0 HC1.0 |  |  |  |
| ***C52A11.2*** | **5** | NA14.0 E18.0 FG2.2 GH2.0 HC31.0 | **5** | NA14.0 E18.0 FG2.2 GH2.0 HC31.0 | **5** | NA14.0 E18.0 FG2.2 GH2.0 HC31.0 | Bm1_46940 | 4 | A14.0 C7.0 E18.0 GH6.0 | contig277.1 | 6 | A14.0 C7.0 E18.0 F7.0 GH6.0 H10.2 |
| ***F19H6.2*** | **3** | AB2.0 E10.1 H12.0 | **3** | AB2.0 E10.1 H12.0 | **3** | AB2.0 E10.1 H12.0 | BM1_21435 | 3 | AB2.0 F3.0 H12.0 | Contig5.100 | 8 | NA35.1 NA85.2 A3.2 D6.2 F3.0 G15.0 H12.0 HC10.0 |
|  |  |  |  |  |  |  | / |  |  | Contig38.62 | 6 | NA6.2 AB2.0 E5.2  F3.0 G15.0 H12.0 |
| ***F21A3.6*** | **4** | NA75.1 B12.2 E5.2 G7.0 | **4** | NA66.1 B12.2 E5.2 G7.0 | **4** | NA74.1 B12.2 E5.2 G7.0 | BM1_41355 | 3 | NA26.1 B12.2 G7.0 | Contig 137.9 | 3 | B12.2 E17.0 G7.0 |
| ***F35B12.8*** | **5** | NA28.2 E17.1 F2.2 H2.0 HC1.0 | **6** | NA28.2 B16.0 E17.1 F2.2 H2.0 HC1.0 | **6** | NA28.2 B16.0 E17.1 F2.2 H2.0 HC1.0 | / |  |  | / |  |  |
| ***F46C8.7*** | **5** | NA45.1 B9.0 F5.1 HC26.0 HC69.0 | 5 | NA46.1 B9.0 F5.1 HC26.0 HC61.0 | 5 | NA46.1 B9.0 F5.1 HC26.0 HC61.0 | Bm1_33065 | 8 | NA7.1 NA39.1 B9.0 E10.0 F5.1 H19.0 HC45.0 HC129.0 | Contig84.53 | 9 | NA154.2 NA172.0 NA196.1  A7.0 B9.0 CD4.2 E10.0 F5.1  GH2.1 |
| ***F49E2.4*** | **4** | A14.2 E8.0 GH30.0 H22.1 | **4** | A14.2 E8.0 GH30.0 H22.1 | **4** | A14.2 E8.0 GH30.0 H22.1 | / |  |  | contig14.29 | 6 | A14.2 E8.0 FG1.2 G16.0 GH26.0 H22.1 |
| ***F52A8.4*** | **5** | NA20.2 NA59.0 A6.0 E14.0 G10.0 | **5** | NA20.2 NA59.1 A6.0 E14.0 G10.0 | **5** | NA20.2 NA59.0 A6.0 E14.0 G10.0 | / |  |  | Contig168.42 | 3 | B11.0 E14.0 G10.0 |
|  |  |  |  |  |  |  | / |  |  | contig326.1 | 5 | NA19.0 NA53.0 E14.0 G10.0  H7.0 |
|  |  |  |  |  |  |  | / |  |  | contig2.166 | 4 | B11.2 E14.0 G10.0 H7.0 |
|  |  |  |  |  |  |  | / |  |  | contig2.164 | 4 | B11.2 E14.0 G10.0 H7.0 |
|  |  |  |  |  |  |  | / |  |  | contig82.11 | 4 | B11.2 E14.0 G10.0 H7.0 |
| ***F56C4.3*** | **4** | C1.0 E14.0 G1.2 H21.0 | **6** | NA11.0 C1.0 E14.0 G1.2 H21.0 HC33.0 | **4** | C1.0 E14.0 G1.2 H21.0 | Bm1_37235 | 5 | A5.0 C3.0 G5.2 H21.0 HC34.1 | / |  |  |
| ***R01E6.6*** | **7** | NA31.0 NA 80.2 B9.0 E16.0 H1.0 HC27.0 HC55.0 | **7** | NA31.0 NA 80.2 B9.0 E16.0 H1.0 HC27.0 HC97.1 | **7** | NA31.0 NA80.2 B9.0 E16.0 H1.0 HC27.0 HC97.1 | Bm1_31825 | 5 | NA27.2 C6.2 E16.0 H1.0 HC38.0 | contig38.51 | 8 | NA5.2 B9.0 E16.0 FG2.1 H1.0 HC2.2 HC25.0 HC54.2 |
| ***R102.9*** | **3** | A14.2 G6.2 H22.1 | **3** | A14.2 G6.2 H22.1 | **3** | A14.2 G6.2 H22.1 | / |  |  | / |  |  |
| ***R11H6.3*** | **6** | NA87.0 C4.0 D3.2 GH1.0 HC10.2 HC37.2 | **5** | NA26.0 C4.0 D3.2 GH1.0 HC37.2 | **6** | NA87.0 C4.0 D3.2 GH1.0 HC10.2 HC37.2 | / |  |  | / |  |  |
| ***R13A1.8*** | **5** | NA37.0 NA81.0 A4.0 FG4.0 H13.2 | **5** | NA37.0 NA81.0 A4.0 FG4.0 H13.2 | **5** | NA96.0 NA140.0 A4.0 FG4.0 H13.2 | Bm1_04635 | 4 | NA17.0 A4.0 E19.0 FG3.0 | / |  |  |
| ***R90.5*** | **6** | NA 22.0 NA46.2 NA82.0 NA119.0 NA142.1 H22.0 | **6** | NA 22.0 NA46.2 NA82.0 NA119.0 NA142.1 H22.0 | **6** | NA 22.0 NA46.2 NA82.0 NA119.0 NA142.1 H22.0 | / |  |  | Contig42.55 | 10 | NA28.1 NA42.0 NA65.1 B2.1  CD4.0 E20.2 F4.0 G9.1 H3.0  H22.0 |
| ***T06A1.3*** | **4** | A3.2 B12.2 E14.0 FG2.0 | **4** | A3.2 B12.2 E14.0 FG2.0 | **4** | A3.2 B12.2 E14.0 FG2.0 | / |  |  | / |  |  |
| ***T22C1.2*** | **4** | A5.0 E18.1 FG9.0 H18.1 | **4** | A5.0 E18.1 FG9.0 H18.1 | **4** | A5.0 E18.1 FG9.0 H18.1 | / |  |  | / |  |  |
| ***W01C9.5*** | **3** | B13.0 EF4.0 HC22.2 | **3** | B13.0 EF4.0 HC22.2 | **3** | B13.0 EF4.0 HC22.2 | / |  |  | / |  |  |
| ***Y15E3A.2*** | **4** | NA23.0 A9.2 G1.2 NA14.2 | **3** | NA25.0 A9.2 G1.2 | **4** | NA24.0 A9.2 G1.2 NA15.2 | / |  |  | / |  |  |
| ***Y17G7B.6*** | **5** | NA12.2 A10.0 C5.2 FG1.0 H21.0 | **5** | NA12.2 A10.0 C5.2 FG1.0 H21.0 | **4** | A10.0 C5.2 FG1.0 H21.0 | / |  |  | / |  |  |
| ***Y22D7AR.5*** | **6** | AB1.2 D3.2 E14.0 G1.2 GH6.2 HC10.0 | **6** | AB1.2 D3.2 E14.0 G1.2 GH6.2 HC10.0 | **6** | AB1.2 D3.2 E14.0 G1.2 GH6.2 HC10.0 | / |  |  | Contig50.31 | 6 | NA11.1 NA38.0 G1.0 GH6.2  H11.0 HC6.0 |
| ***Y57G7A.9*** | **3** | B9.0 E15.0 G2.0 | **3** | B9.0 E15.0 G2.0 | **3** | B9.0 E15.0 G2.0 | / |  |  | / |  |  |
| ***Y58A7A.6*** | **3** | NA28.0 NA85.1 F3.0 | **3** | NA28.0 NA85.1 F3.0 | **3** | NA28.0 NA85.1 F3.0 | / |  |  | contig106.6 | 5 | A13.0 E3.0 F3.0 H1.0 H26.0 |
| ***Y75B7AL.1*** | **6** | NA35.2 NA134.0 NA372.2 CD2.0 EF10.0 FG7.0 | **6** | NA34.2 NA133.0 NA370.2 CD2.0 EF10.0 FG7.0 | **6** | NA35.2 NA133.0 NA371.2 CD2.0 EF10.0 FG7.0 |  |  |  | Contig94.7 |  | NA24.0 NA67.0 NA93.2 B9.0  CD9.1 E6.0 EF10.0 H1.1 |
| ***ZK637.13*** | **1** | E3.2 | **1** | E3.2 | **1** | E3.2 | Bm1_50430 | 3 | B12.2 EF2.1 G7.0 | contig14.30 | 5 | B12.2 E3.2 EF7.0 G7.0 H15.1 |
