# Supplementary material for: The Caenorhabditis globin gene family reveals extensive nematode-specific radiation and diversification
Source: BMC Evol Biol. 2008 Oct 9;8:279. doi: 10.1186/1471-2148-8-279 (PMC2576238; doi:10.1186/1471-2148-8-279)
Supplement: Additional file 2 — Manual alignment of nematode globins. [file 1471-2148-8-279-S2.doc]

**Additional file 2**

helices : -aaaaaaaAaaAAaaAa-----------------bbbbbBbbBBbbBBbbcCccccc----------------D-------------------ddddddde
 : --------|--||--|-----------------------|--||--||---|---------------------|--|------------------------
ZK637.13 : MSMNRQEISDLCVKSLEG---------RMVGTEAQNIENGNAFYRYFFTNFPDLRVY----------------FKGA-------------EKYTADDVKKS
CBG0687 : MSMTRQEIQDLCVKSLEE---------KMVGTTDKGIANGNGFYQYFFTNFPDLRVY----------------FKGA-------------EKFTAEDVKKS
CRZK63 : MSMTREEIRDLCVKSLEE---------KMVGTEEKNVDNGNGFYRYFFTNFPDLRVY----------------FKGA-------------EKYTAEDVKKS
F49E2.4 : ITDEEVTAIRDVWRRAKT------------------DNVGKKILQTLIEKRPKFAEY----------------FG-I-----------QSESLDIRALNQS
CBG16082 : MSDEEVSAIREVWIRAKT------------------DNVGKKILQTLIEKRPKFAEY----------------FG-I-----------QSESLDIRALNQS
CRF49 : MSDEEVAAIRDVWVRAKT------------------DNVGKKILQTLIEKRPKFAEY----------------FG-I-----------QSESLDIRALNQS
C52A11.2 : LNKKDRTLLRETWQRLDD----------------PKDIVGLIFLDIVNDIEPDLKKV----------------FG-V-------------DRAPRAAMLKM
CBG03023 : LNKKDRTLLRETWQRLEE----------------PKDIVGLIFLDIVNDIEPDLKKV----------------FG-V-------------DRAPRAAMLKM
CRC52 : LNKKDRTLLRETWQRLED----------------PKDIVGLIFLDIVNDIEPDLKKV----------------FG-V-------------DRAPRAAMLKM
C28F5.2 : LTFSQKQALNLSWRLLPQ-----------------ASACFRKIFLELEIASPKVKQIFYKAALVDA-------FNK--------------------DDDNS
CBG13047 : LTFSQKQALNLSWRLLKP----------------QASACFRKIFLELEIASPKVKQIFYKAALVDA-------FNK--------------------DDDNT
CRC28 : LTFSQKQALNLSWRLLKP----------------QASTCFRKIFLELEIASPKVKQIFYKAALVDA-------FNK--------------------DEDNS
W01C9.5 : LTPSQVSVVRRSWRHINT---------------KGLIIVLTRCFSRLESNCPIVSQC----------------FQSA-------------TYSLSTNPNGV
CBG00571 : LSPSQVSVIRRSWRHINT---------------KGLITVLTRCFSRLESNCPIASQC----------------FQSA-------------TYSLSTNPCGV
CRW01 : LTPSQVSVVRRSWRHINT---------------KGLIIVLTRCFSRLESNCPIVSQC----------------FQSA-------------TYSLSTNPNGV
C29F5.7 : LNAKTKKLVIQEWPRVLA----------------QCPELFTEIWHKSATRSTSIKLA----------------FGIA-----------ENESPMQNAAFLG
CBG02622 : LNAKTKKLVIQEWPRVLA----------------QCPELFTEIWHKSATRSTSIKLA----------------FGIA-----------ENESPMQNAAFLG
CRC29 : LNAKTKKLVIQEWPRVLA----------------QCPELFTEIWHKSATRSTSIKLA----------------FGIA-----------ENESPMQNAAFLG
F19H6.2 : LTRRERILLEQSWRKTRK---------------TGADHIGSKIFFMVLTAQPDIKAI----------------FG-L-------------EKIPTGRLKYD
CBG00138 : LTRRERILLEQSWRKTRK---------------TGADHIGSKIFFMVTAQ-PDIKAI----------------FG-L-------------EKIPTGRLKYD
CRF19H6.2 : LTRRERILLEQSWRKTRK---------------TGADHIGSKIFFMVLTAQPDIKAI----------------FG-L-------------EKIPTGRLKYD
R01E6.6 : ISAQGREIITQCFENPHS-------------------EFANKVVQRIFEKR-EDYQKYIMN------------L-------------------GKERSSIV
CBG07422 : ITSQCREIISQCFDNPHS-------------------EFANKVVQRIFEKR-EDYQKYIMN------------L-------------------GKERSVIV
CRR01 : ISAQGRDIISQCFDNPHS-------------------EFANKVVQRIFEKR-EDYQKYIMN------------L-------------------GKERSVIV
F46C8.7 : LSKIQKRAIRFTWHRLQT-----------RNGGKRVENVFEEVFDKLVKNLPNIRDM----------------FST-------------RMFLCAMSRGTT
CBG16720 : LSKIQKRAIRFTWHRLQT-----------RNGGKRVENVFEEVFDKLVKNLPNIRDM----------------FST-------------RMFLCAMSRGTT
CRF46 : LSKIQKRAIRFTWHRLQT-----------RNGGKRVENVFEEVFDKLVKNLPNIRDM----------------FST-------------RMFLCAMSRGTT
R13A1.8 : IDKESCEVVADSWRLVES----------RSSAAETSACFGLFVFQRVFSKIPMLRPL----------------FG-L------------SESDDVFDLPDN
CBG05809 : IDKECCEIIGDSWRIVES----------RASSTFPTACFGLFVLRRVLQRIPILCPL----------------FS-L------------SESDDIFKLPEN
CRR13 : IDKESCEVISESWRIVES----------RSASSIPTSCFGLFVFRRVLSKIPMLCPL----------------FS-L------------SESDDIFNLPES
C06H2.5 : LSPHQVQLLTSTWPRIKT-----------------QSSLFTQVFKVLMQRSPVCREM----------------FQKM-------------SIVGGFSSNSV
CBG23115 : LSPHQVQLLTSTWPRIKT-----------------QSHLFTQVFKVLMQRSPVCREM----------------FQKM-------------SIVGGFSSNSV
CRC06H2.5 : LSPHQVQLLTSTWPRIKT-----------------QSTLFTQVFKVLMQRSPVCREM----------------FQKM-------------SIVGGFSSNSV
C26C6.7 : LTCAQIHLVRALWRQVYT--------------TKGPTVIGASIYHRLCFKN-VMVKEQ---------------MKQV--------------ELPPKFQNRD
CBG11881 : LTCAQIHLVRALWRQVYT--------------TKGPTVIGASIYHRLCFKN-LMVKEQ---------------MKQV--------------ELPPKFQNRD
CRC26C6.7 : IEPVLCFDDEVSVSTRRL---------------SASEKARQNVIAQRRQSN-VQVKEQ---------------MKQV--------------ELPPKFQNRD
F52A8.4 : PNVYEKELLRRTWSDEFD----------------NLYELGSAIYCYIFDHNPNCKQL----------------FPFI-------------SKYQGDEWKES
CBG11915 : PNVYEKELLRRTWSDEFD----------------NLYELGSAIYCYIFDHNPNCKQL----------------FPFI-------------SKYQGDEWKES
CRF52 : PNVYEKELLRRTWSDEFD----------------NLYELGSAIYCYIFDHNPNCKQL----------------FPFI-------------SKYQGDEWKES
T22C1.2 : LNSYQKSIVRNAWRHMSQ---------------KGPSNCGSTITRRMMARKSTIGD-----------------IL------------------------DR
CBG08252 : LNSYQKSILRNAWRHMSQ---------------KGPSNCGSTITRRMMARKSTIGD-----------------VL------------------------DR
CRT22 : LNSYQKSIIRNAWRHMSQ---------------KGPSNCGSTITRRMMARKSTIGE-----------------VL------------------------DR
C18C4.1 : IVDDDFELARTHWIQLQK--------------SNKQGLAIRGCFLTMLEKYPQVRPIWG--------------FGK---------RIEGRGDETWKPEIVE
CBG09371 : IVDDDFELARAHWIQLQK--------------SNKQGLAIRGCFLTMLEKYPQVRPIWG--------------FGK---------RIEGRIDETWKPELVE
CRC18C4.1 : IVDDDFELARTHWIQLQK--------------SNKQGLAIRGCFLTMLEKFPQVRPIWG--------------FGK---------RIEGRVEETWKPEIVE
R11H6.3 : LKHEHIRALKTTWARLCE-------------PPRANCKGIVSLVERVWEKL-DTKDKDVRNI-----------FYNA------AFVDSMHERCERRRSGSI
CBG04577 : NLKHEHIRALKTTWARLC------------EPPRANCKGIVSLVERVWEKL-DTKDKDVRNI-----------FYNA------AFVDSMHERCERRRSGSI
CRR11 : NLKHEHIRALKTTWARLC------------EPPRANCKGIVSLVERVWEKL-DTKDKDVRNI-----------FYNA------AFVDSMHERCERRRSGSI
C23H5.2 : FSQEEKDILRRSWKVLDK----------------NLNHTAYNIFEMIFNQSPDTRQL----------------FPFM----------------KFNTGGRS
CBG10551 : FSPEEKDIIRRSWKVLDK----------------NLNNTAYNIFEMIFNQSPDTKQL----------------FPFM----------------KFQQSGKS
CRC23 : FSPEEKDSLRRSWKVLDK----------------NLNNTAYNIFEMIFNQSPDTKQL----------------FPFM----------------KFNTSGRS
CBG24799 : DSYRDFFTLKNWWKTVDR----------------KRVEASGYMFSKYLNDFPENKDL----------------YPKL----------KNVKAATVDMNCSD
CRT06 : DSYRDFFTLKNWWKTVDR----------------KRVEASGYMFSKYLNDFPENKNF----------------YPKL----------KNVNAATVDMSCSD
T06A1.3 : DSYRDFFTLKNWWKSVDR----------------KRVEASTYMFSKYLNDFPQNKDL----------------YLKL----------KNVNAQTVDMNCSD
Y17G7B.6 : LSVKQKKLLRQSFNAMNS--------------GGTFLKLMEKIFRRLETKCPDMRSI----------------FLT--------TAFVNSLSRERQTPPLV
CBG21021 : LTAKQRKLLRQSFNAMNS--------------GGTFLKLMEKIFRRLENKCPDMRSI----------------FLT--------TAFVNSLSRERQTPPLV
CRY17 : LTPKQRKLLRQSFNAMNS--------------GGTFLKLMEKIFRRLENKCPDMRSI----------------FLT--------TAFVNSLSRERQTPPLV
Y57G7A.9 : RPKLDIDRVRSVWMDHIN----------------GNDQYFQEVIHRICKRN-EGIRCA---------------MLAP------NAQHAESVAEEDFVLSNI
CBG07112 : RPKLDVERIRTVWMEHIN----------------GNDDYFQEVIRRICKRN-EGIRCA---------------MLTQ------NAQHAESVAEEDFVLSNI
CRY57 : RPKLDVERIRAVWMDHIN----------------GNDDYFQEVIHRICKRN-DGIRCA---------------MLTQ------NAQHAESAAEEDFVLSNI
Y15E3A.2 : ISPEHQKLIKRSWNRIPK---------------AQFGRASLEAFITAAQV-THAI------------------FVD------------------------K
CBG07681 : LVPEQQRLIRQSWNRIPK---------------WQFAKSVLTAFFRACHA-PQLI------------------FPN------------------------Q
CRY15 : LVPDHHKLIRKSWGRIPK---------------TQFGKAALEAFIRISEV-NHSI------------------FGD------------------------K
Y58A7A.6 : LSRDDKRIIETCWFKCSQ---------------KQLRKCSCDMFWDILHTD-EDILRL---------------FR-L-------------DHVAPNRLKDN
CBG08670 : LTRDDKRVIETCWFKCSQ---------------KQLRKCSCDMFWDILHTD-EEILRL---------------FR-L-------------DHVAPNRLKEN
CRY58 : LTRDDKRIIETCWFKCSQ---------------KQLRKCSCDMFWDILHTD-EDILRL---------------FR-L-------------DHVSPNRLKEN
C18C4.9 : LTQPQILFVRKTWNHARN---------------QGALEPAISIFRNSFFKNPEIRQMIM--------------FGT--------------------KNEGH
CBG09371 : LTQPQILFVRKTWNHARN---------------QGALEPAISIFRNSFFKNPEIRQMIM--------------FGT-------------------KNEGHE
CRC18C4.9 : LTQPQILFVRKTWSHARN---------------QGALEPAISIFRNSFFKNPEIRQMIM--------------FGT--------------------KNEGH
R90.5 : LSPYQQKLLVQCWPNIYT--------------TGASGPFANSLYSTLSSRNAKAKELLAKADGVAV-------FSK----------------------SDF
CBG09511 : LTAYQQKLLTQSWPNIFT--------------TGASGPFANSLFATLSARNAKAKELLTKANGVAV-------FAK----------------------SDM
CRR90.5 : LTAYQQKLLVQSWPNIYT--------------TGASGPFANSLYSTLSARNAKAKELMTKANGVAM-------FSK----------------------SDM
C36E8.2 : LSVNQRQIIKGCMDNSKD-------------------DLGERIFRRALERR-DDFKQ----------------FI-------------------------D
CBG03635 : LSVNQRQIIKGCMDNSKD-------------------DLGERIFRRALERR-DDFKQ----------------FI-------------------------D
CRC36 : LSISQRQIIKGCVDNSKD-------------------DLGERIFRRVLEKR-DDFKQ----------------FI-------------------------D
Y75B7AL.1 : LLGDRLSILKSSWEKANE---------------MTNGEIGVRVAWNMVRKHPNLCKNDEPEK-----------VSLL--------------NGSCKRSIDH
CBG06424 : LLGDRLSILISSWEKANE---------------MTNGEIGVRVAWNMVRKHPTMCKKTLPEPEK---------VSLL--------------NGSCKRSIDH
CRY75 : LLGDRLSILISSWEKAKE---------------MTNEEIGVRVAWNMFRKHPTMCKKDEPEK-----------VSLL--------------NGSCKRSIDH
F35B12.8 : CEQEEVNKMIESYQKIDD-----------------KYALFEQMFLTIFLEQ-EVEMAYS--------------FG-L-------------ENLNEQQLKVE
CBG23302 : CEQEEVNKMIESYQKIDD-----------------KYALFEQMFLTIFLEQ-EVEMAFS--------------FG-L-------------ENLNEQQLKVE
CRF35B12.8 : CEQEEVNKMIESYQKIDD-----------------KYALFEQMFLTIFLEQ-EVEMAFS--------------FG-L-------------ENLNEQQLKVE
F21A3.6 : LSDRQRDVLQKTFAPILQ----------------DCVRNGLKIFVRLFSEYPRYKLIWPQ-------------F----------------RAIPDSSLMNA
CBG18593 : LSDRQREILQKTFTSIEQ----------------DCVRNGLKIFVRLFAEYPRYKLIWPQ-------------F----------------RAIPDSSLMNA
CRF21A3.6 : LSDRQRDILQKTFTTIEQ----------------DCVRNGLKIFVRLFAEYPRYKLIWPQ-------------F----------------RAIPDSSLMNA
C06E4.7 : LSVAERQCICASWEKAST-----------------QSDIGCELVARLLNDN-RTRFRALLECKSGS-------FLG-------------SGNYTTEDVNGM
CBG05824 : LSIAERQVICASWEKAST-----------------QSDIGCELVARLLNDN-RTRFRALLECKSGS-------FLG-------------SGNYTTEDVNGM
CRC06E4.7 : LSIAERQCICASWEKAST-----------------QSDIGCELVARLLNDN-RTRFRALLECKSGS-------FLG-------------SGNYTTEDVNGM
F56C4.3 : FTQEEKNDLEHSWNLVEG----------------KKNHIACDIYEMIFNQCPEARRL----------------FPKL-------------KFVGSKPDRKN
CBG04428 : FTQEEKNDLEHSWNLVEG----------------KKNHIACDIYEMIFNQCPEARRL----------------FPKL-------------KFVGSKPDRKN
CRF56 : FTQEEKNDLEHSWSLVEA----------------KKNHIACDIYEMIFNQCPEARRL----------------FPKL-------------KFVGSKPDRKN
R102.9 : LTTDEMQAVRDAWKRAKE------------------REIGKHILRALIERKPQ--------------------FKDY---------FGIHVDEKNHDVYSC
CBG17640 : LSSDEMQAVRDSWKRAKE------------------REIGKHILQALIERKPQ--------------------FKDY---------FGIHVDEKKDDVFSC
CRR102 : LSSDEMQAVRDSWKRAKE------------------REIGKHILQALIERKPQ--------------------FKDY---------FGIHVDEKNDDVFSC
C09H10.8 : DPQLLSDAISEAWLKSAE--------------------LTPTWVWALVDL-PETMHATYKENAQ---------FLNI--------------------INQV
CBG02965 : STHRKHVVHTEAWLKSAE--------------------LTPTWVWALVDL-PETMHSRYKENAQ---------FLNI--------------------INQV
CRC09 : DPQLLNDAISEAWLKSAE--------------------LTPTWVWALVDL-PETMHATYKENAQ---------FLNI--------------------INQV
Y22D7AR.5 : LTPIDREILNKSWGIVSK----------------DMQQVAVNIFQMIFEQAPDAKLM----------------FSFM--------------MKDYKEDKKS
CB_Y22 : LTPIDREILNKSWGIVSK----------------DMQQVAVNIFQMIFEQAPDAKLM----------------FSFM---------------MKDYKEDKK
CR_Y22 : LTPIDREILNKSWAIVSK----------------DMQQVAVNIFQMIFEQAPDAKLM----------------FSFM--------------MKDYKEDKKS
Pp27.56 : ----ETHLARAHWILLHK--------------MNKQGTVIQSTFEHLMTEFKHTRPI----------------WQF-------GRNIDENVKDWNKELHED
Pp42.55 : LTAYQQKLLIQCWPNIYS--------------TGPGGQFASAIYNRLQNSCPKAKQL----------------LAKA-------------NGVAVFANSDV
Pp10.104 : LTFPQIHLIRTLWRQIYM--------------TKGPTVIGTTLFHRLCFKSPEIKDQ----------------IRAA--------------PLPAQFNNHD
Pp94.7 : AFNRDRYIVLNSTWEQAK--------------KLTNDQIGYTVLYSMIRKQPSLLDPLRPPSFAPGGE-----THIV--------TLIGLPVKRSFDFMAQ
Pp50.31 : LIEGILVQTRNAYSDYGV--------------PSPFILHSLSLLESVALPHFYKHSH----------------NINI-----------PMFNHLGWLVEKW
Pp106.6 : LTPEQKRILETSWVKATP---------------KQIRKATEDVFASIINHDRSLAVM----------------FRL--------------DDVPINRIREN
Pp68.42 : ILAHSPELFMCAWQRSAG-----------------RSASIKKTFVNVDASDDEVKIG----------------FSEL----------------SGVIQNFF
Pp14.29 : LTDEEVAAVRNVWIRAKT------------------EDIGKKILQTLIEKRPKFAEY----------------FGIL----------CQSDKLDMNSLKES
Pp84.53 : LNKMQKKALRFTWHRLQT-----------RNGGKRVENVFEEVFDKLVRQLPCIRDM----------------FTTR-------------TFLCAMSKAEM
Pp68.66 : LSSKTKKRVMKEWKNVMA----------------HDPLLFQKAWVGSANRSMSIKQI----------------FGIP----------------LDQDAGEN
Pp168.42 : STPEDKKLMEKTWSEEFD----------------VLLTLGSDIYNYIFKNMSACKRL----------------FPWI-----------IKYEDEGVDWKKT
Pp326.1 : SNARDQALFRKTWSDDFE----------------VLFSIGSSIYMSVLNRCSTLEFSFSHAFEGPHGTACKSLFPWV-----------AKYEKAGRNYAEQ
Pp2.166 : QNPADTALLQKTWTDDFE----------------VLFSLGSKMYITAFEGAHGAACKSL--------------FPWV-----------ERYEKAGRNYAEQ
Pp2.164 : TNESDQALIKATWSEDFE----------------TLYLLGSKMYLQIFAQDATIKAL----------------FPWI-----------AQYEKAGRDFTLE
Pp82.11 : SNPQDTALLRRTWTDDFE----------------VLYSIGSNIYLNVF--NGECGAAAKAL------------FPAF-----------ARYEAEGLNYAEQ
Pp360.2 : LNASQRSIIKYCLDNAK-------------------EDMADRIVRRVGEKKDDFKA-----------------FIEA---------------------LPK
Pp14.30 : MSVEVARLCKHSLESARV------------GTDEDKVQNGRDFYKFFFTNYPDLRVY----------------FKGA-------------EKFTADDVQKS
Pp38.62 : MPRRDRLLIVKSWRKTQK---------------TGAEHIGSKIFLRVLTTQPDIKAI----------------FGL--------------EKIPQGRLKYD
Pp5.100 : MHRRDRLLISKSWMKAQK---------------TGAEYIGAKIFHRLLTAQPEIKTI----------------FGL--------------------EHIPH
Pp277_1 : LNKKDRTCLRESFQFQRL--------------QEPKEIVGLIFVDIVNDIEPDLRTV----------------FGV--------------NRAPKAAMLSM
Pp43.86 : EFLARFAVLLTAPGHPGV-----VEAHSSTEVAELANSTFRKIFLELEIACPKVKQI----------------FYKA-----------ALVDAFNKDGENA
Pp94.50 : QTGSRRNSRRSSAVGLPQ-----------ISTSCALLRKCITGERMLTFKSPTAREI----------------FQKM-------------SIVEGFKRQSS
Pp137.9 : LDARNRQLIRDSFEFMRA----------------EKNRNGLGIFIRLFAEFPQYKNI----------------WSNF-------------RAIPDSSLISS
Pp38.51 : ITANGREIIVQCFENPH-------------------SEFGNKTCQRIFEKRPDFQNYV---------------FAL-----------------GKERAFQM
Bm1_37235 : MIFDQCPEARQLFSFMNL--------------DLNNCKKKNNDFVFQALRFIQKSYEKHD-------------FMTL--------------YYWKELLEKA
Bm1_31825 : ISPQGREIIQACFGNHH-------------------NEIGYRICMRVFEKRTDYQRF----------------VYAL----------------GREKWLSA
Bm1_41355 : LSLEDRKLLHETFNLFEK--DLTTNGLRIFLQLALITKFIYLFCMRTLSENPDYKYFWPQ-------------F----------------RAIPDSSLISS
Bm1_13755 : LVLSQRQIVKGCMDKAK-------------------DDIAERIYRRIIEXRDDFRK-----------------FVEA---------------------LSE
Bm1_27145 : ------SIILFCMENAR-------------------SDIALRIVQRMAHKRDDFAQ-----------------FYGN---------------------LSN
Bm1_17795 : LSSYQIHLLQQSWQRLRC-----------------SPNFFINVFRTVISKNTIAKEL----------------FRK-------------TSIIDGFTSYKC
Bm1_02240 : LTSAQIHLIRNIWRQVYI--------------TKGPTVIGSTLLHGIYFKSKKIKDQ----------------FFR--------------CPFPHRFPNRD
Bm1_33065 : NRLQKRFLRFTWHRLQTK------------NGGKRVGNVFEEVYERLLRQLPGTWEM----------------FTT-------------RTFLSAMSRSET
Bm1_21435 : LSKSQRITIENSWKRATK--------------SNAREQVGIQLFGRILTARPEMKHL----------------FGL--------------QKIPEGRLKYD
Bm1_50430 : --MSRLVIKAKCMKVLNE--------AGRVGTDDEAIQHGKNFYKFMFGHHPDLRVF----------------FKGAENFTPADVQNSDRFAKQGNKNSSL
Bm1_46940 : FVKKERTCLRESFQKLED----------------PKEIIGIIFVKILNDIAPELKKP----------------FGV--------------ERSPKATMPKM
Bm1_24705 : LTIHQQQALLTSWKSLRP----------------IIQTLMRKILNNLEEEVPKVKQI----------------FCQT---------AVLDAFNRESTSENP
Bm1_04635 : LSEIQQELIRQSWQTISA------------KLEVNEQNFGFFVYRRVFEHNPLLKRA----------------FHVE-------------EYDLLDSIPRE
Gossypium : FTEEQEALVVKSWTVMKK----------------NAAELGLKFFLKIFEIAPSAKKL----------------FSFL--------------RDSNVPLEQN
Malus : FTEEQETLVVKSWGVMKK----------------NAAELGLKFFLKIFEIAPSAQKL----------------FSFL--------------RDSDIPLEKN
Glycine : FSEEQEALVVKSWNVMKK----------------NSGELGLKFFLKIFEIAPSAQKL----------------FSFL--------------RDSTVPLEQN
Alnus : FTEEQEAVVVKSWNAMKP----------------NAGELGLKFFLKIFEIAPSAQKL----------------FSFL--------------RDSNVSLERN
Citrus : FTEEQEALVVKSWNAMKK----------------NSGELALKFFLRIFEIAPSAKKL----------------FTFL--------------KDSDIPVEQN
Nemvec141000032 : -SEAQKYLVRETWETIEP----------------QKQTVGKKAFLRFFDMNPDYQNL----------------FPEF-------------KSLSYEELQKA
Nemvec3000224 : -STRRKKLVRESWELIEP----------------VKITIGKRLFTRLFDVNPNMQDT----------------FPNF-------------KGKELKDILNS
Nemvec50000067 : -DAKETQLVRKTWAILGD----------------RQVEVGKSLFLRFFEEHPTSKDL----------------FPEF-------------RNISNEKIAES
Nemvec7000121 : -TERQIKLVQDTWRLLIP----------------SQKKTAMIFYLKLFTLDPIFKEV----------------FS-F-------------HTENEGQLEQD
Nemvec76000030 : -TETQKYYIKQSWMGLES----------------NKGELGIEIFLRLFSENPTLQLM----------------FPEF------------REYSTLEELKES
Paragonimus1 : MAPLTQAEVDGVVSELNPFLASDA----------KKVELGLGAYKALLTAKPEYIQL----------------FSKL-------------HGLTIDNVFQS
Paragonimus2 : MAVLTQGEVDSLLAELGPRME-------------NLEEFGMSVYKELFTAHPEYISL----------------FSKL-------------QGLTLDNVMQS
Clonorchis : MAPLSKDEVDALFEELNPLVSTTE----------QRTEFGKAVYMALFSAYPEYIQL----------------FTKM-------------QGLTKDNVEAS
Isoparorchis : -MVLTKDEFDSLLHELDPKIDTEE----------HRMELGLGAYTELFAAHPEYIKK----------------FSRL-------------QEATPANVMAQ
Paramphistomum : -MTLTKHEQDILLKELGPHVDTPA----------HIVETGLGAYHALFTAHPQYISH----------------FSRL-------------EGHTIENVMQS
Schistosoma : MAAVTQSQVDHLITELEPHVDTEA----------HKLELGLKVYECFLKDRPEYICK----------------FSRL-------------QGLDASNVAQS
Pseadoterranova : HSVRDHCMNSLEYIAIGDKE--------------HQKQNGIDLYKHMFEHYPHMRKA----------------FKGR-------------ENFTKEDVQKD
Heterorhabditis : SIEDVKMHCKASMETIPIGTGE------------KELQNGKDFYKYFFANYKDLRKY----------------FKGA-------------KNYSPDDVQNS
Mermis_Eye : -MVVNLDILRAQLAKLP-----------------INEFNGPKFYVHMFSSQPDWRNY----------------FKGS-------------EAIKPEEVPTC
Mermis_Body : -MVVNLDIIRAQLAKLP-----------------INEINGPKFYVHMFSTQPDLRNF----------------FKGS-------------ENIKPEEVPAS
Toxoperient : SAETKRELCMKSLKDVHVGTGD------------HAKQCGIDLYKHMFEHYPDLRKF----------------FKNR-------------ENYTAEDVQKD
GRP00348_1 : ANLSPISANAREIIQFCFDN--------------PHNEIGSRICSRLLEKRPDFRQ-----------------F---------------------AYSMGK
ACP03829_1 : --------TAASLSVAGLGKTP------------DKVQNGKDFYKYFFTHHPEHRKY----------------FKGA-------------ENFTADDVQKS
ACP01487_2 : --------LETVAIFPGP----------------KEVQNAKDFYKYMFTHHPDLRRY----------------FKGA-------------ENFTAEDVQKS
ACP00369_1 : FTNEEKEVLLHSWKVLEP----------------HKQALGCDIYEMIFNQCPEARKL----------------FPKM-------------KFVNSKPDKKA
ALP00043_1 : --RDIATTYVKSVGTVQCGTCQ------------KTIANGTEFYALLFDKHPHLRHY----------------FKGN-------------ENLTGTDVKKN
AscarisMb : ----MATACVKSLESVQCGTCE------------KTIANGTEFYALLFDKHPDLRHY----------------FKGN-------------ENLTGADVKKS
AscarisHb : -ANKTRELCMKSLEHAKVDTSN------------EARQDGIDLYKHMFENYPPLRKY----------------FKNR-------------EEYTAEDVQND
Syng_Intracell : TPCEVKKHVKNSLEYAPIGKTP------------EEIQNGKDFYKHMFTHHPDLRRY----------------FKGA-------------ESFTADDVQKS
Syng_Cuticul : AAADVKKHVVDSLVNVPL----------------GNDQTGKDFYKYFFTNHPDLRKY----------------FKGF-------------ETFTADDVQKS
ASP00019_1 : RELCMKSLEHAKVDTSN-----------------EARQDGIDLYKHMFENYPPLRKY----------------FKNR-------------EEYTAEDVQND
ASP00780_1 : SLAEVKASCMKSLEKARIGTDE------------GAIQDGKDFYKYMFGHYPDLRIY----------------FKGA-------------EKFTPDDVQRS
ASP17423_1 : ------NHLESAQCGTCD----------------KAIENGTGFYALLFDKHPELRHY----------------FKGN-------------ENLTGAEVKKS
AYP02881_1 : LSFSQKQALTASWRLLRP----------------QAPGLFRKVFLELEIVSSKVKQI----------------FYKA-----------LCVDAFNKDEENI
AYP03578_1 : APADVKKHTVASLAVAGLGKTP------------DKVQNGKDFYKYFFTHHPENRKY----------------FKGA-------------ENFTADDVQKS
AYP00272_1 : APADVKKHTVASLSVAGLGKTP------------DKVQNGKDFYKYFFTHHPEHRKY----------------FKGA-------------ENFTADDVQKS
AYP01816_1 : APADVRRHTVASLANVPL----------------MKEHHGRDFYKFFFTTYPEHRQY----------------YKGA-------------ENFNADEVEKS
AYP00544_1 : APADVRKHTVASLVNVPV----------------MKEHHGRDFYKYFFTTHPEHRRY----------------YKGA-------------ENFNADDVEKS
DIP00455_1 : LSKSQRATIENSWKRATMSN--------------AREQVGIELFGRILTARPEMKHL----------------FGL--------------QKIPEGRLKYD
HCP00759_3 : -MADVKKACLESLKVIPLGRTP------------AETQHGTDFYKYLFGHHPDLRKY----------------FKGA-------------ENFTPDDVQKS
HCP00759_2 : -MADVKKACLESLKVIPLGRTP------------AETQHGTDFYKYLFGHHPDLRKY----------------FKGA-------------ENFTPDDVQKS
HCP00759_1 : -MADVKKACLESMKVIPLGKTP------------AETQHGTDFYKYLFGHHPDLRKY----------------FKGA-------------ENFTPDDVQKS
HCP00786_1 : -MADVKKACMESLKVVPLGKTP------------AEIQNGTDFYKYLFGHHPDLRKY----------------FKGA-------------ESFTPDDVQKS
HCP13111_1 : SPEDFPKNAVAALEHAPLGTAP------------EKDHIGRDFYKHYFTKHPEVRKY----------------FIGA-------------ESITPDEVDKS
HCP00770_1 : GPEDVRKNSLAALESVALGTTP------------EKVQNGKDFYKYLFTEHPEVRKY----------------FKGA-------------ESFTADDVQKS
HCP00770_3 : GPEDVRKNSLAALESVALGTTP------------EKVQNGKDFYKYLFTEHPEVRKY----------------FKGA-------------ESFTADDVQKS
HCP00208_1 : SPEDVKKNAVAALEHAPLGTTP------------EKDHIGRDFYKHYFTKHPEVRKY----------------FIGA-------------ESITPDEVDKS
HCP00202_4 : GSRRVKKNAVAALESVPVGTTP------------DKIQNGKDFYKYFFTKHPELRKY----------------FKGA-------------ESFTADDVQNT
HCP00208_2 : SPEDIKKNAVAALEHAPLGTTP------------EKDHIGRDFYKHYFTKHPEVRKY----------------FIGA-------------ESITPDEVDKS
HCP01314_3 : SPEEVKKNAVAALESVPVGTTP------------EKIQNGKDFYKHFFTHHPENRKY----------------FKGA-------------ESFTADDVQNT
HCP00202_3 : APEDVKKNAVAALESVPVGTTP------------DKIQNGKDFYKYFFTKHPELRKY----------------FKGA-------------ESFTADDVQNT
HCP00333_2 : APEDVKKNAVAALESVPVGTTP------------DKIQNGKDFYKYFFTKHPELRKY----------------FKGA-------------ESFTADDVQNT
HCP00202_2 : LQKKSKRTLWQHWKVFQSGTDFL-----------EKIQNGKDFYKHFFTHHPENRKY----------------FKGA-------------ESFTADDVQNT
HCP00202_1 : SRRCQKERCSLLXESVPVGTTP------------DKIQNGKDFYKYFFTKHPELRKY----------------FKGA-------------ESFTTDDVQNT
HCP00770_2 : GPEDVRKNSLAALESVALGTTP------------EKVQNGKDFYKYLFTEHSLVRKY----------------FKGA-------------ESFTADDVQKS
HCP00202_5 : APEDVKKNAVAALESVPVGTTP------------DKIQNGKDFYKYFFTKHPELLFY----------------FKGA-------------ESFTADDVQNT
HCP02815_1 : SDADVKKNFLAALESAPLGSTP------------EKIQNGKDFYKFLFTNNLFVRKY----------------FKGA-------------ESFTADDVQKS
HCP08501_1 : -MADVRKACFTSMATLPLGTTP------------KDQQSGTDFCKYLFTHHQDLRKY----------------FKGA-------------ENFTGDDVQKS
HGP06223_2 : TREEIQAVCVQSLKQLKIGVGE------------EELQNGKDFYKFFFTNYPTLRVY----------------FKGA-------------EKYEAEDVQKS
MJP04640_1 : KRLEIQSLCLESLSKIKIGTGP------------EEKQNGKDFYKYFFTNYPDLRVY----------------FKGA-------------EKYTADDVQKS
MIP00586_2 : KRLEIQSLCLESLSKIKIGTGP------------EEKQNGKDFYKYFFTNYPDLRVY----------------FKGA-------------EKYTADDVQKS
MHP02564_2 : KRLEIKSLCIESISKLKIGTGL------------EEKQNGKDFYKYFFTNYPDLRVY----------------FKGA-------------EKFTADDVQKS
NAP00088_1 : RIRHEKKHTVASLGVVPVGKTP------------DKIQNGIDFYKYFFTNHPEARKY----------------FKGA-------------ENFTADDVQKS
Nippo_Body : -MADVKKNCLASLSLAPISKA-------------QQAQVGKDFYKFFFTNHPDLRKY----------------FKGA-------------ENFTADDVQKS
Nippo_cuticul : --ADVKKHTVESMKAVPVGR--------------DKAQNGIDFYKFFFTHHKDLRKF----------------FKGA-------------ENFGADDVQKS
NBP00095_1 : -MADVKKHCLASLASVPLGKTP------------DKMQNGKDFYKFFFTHHPDLRKY----------------FKGA-------------ENFTADDVQKS
NBP00124_1 : SPADVKKNAVASLATVPLGKTP------------EKVQNGKDFYKYFFTKHPEHRKY----------------FKGA-------------ESFTADDVQKS
NBP00197_1 : SPADVKKHTVESMKAVPVGR--------------DKAQNGIDFYKFFFTNHKDLRKF----------------FKGA-------------EGFTADDVQKS
NBP00328_1 : SPADVKKHTVESLKAVPVGR--------------DKAQNGIDFYKFFFTNHKDLRKF----------------FKGA-------------EKFSADDVQKS
OOP00190_2 : -----RRTAWAALESVALGTTP------------EKVQNGKDFYKYLFTEHPDVRKY----------------FKGA-------------ESFTADDVQKS
OOP00190_1 : GPEDVKKNSLAALESVALGTTP------------EKVQNGKDFYKYLFTEHPDVRKY----------------FKGA-------------ESFTADDVQKS
OOP03513_1 : -----------VSESRTTRKEL------------AETQHGTDFYKYLFGHHPDLRKY----------------FKGA-------------ENFTPDDVQKS
OOP03348_2 : SDEDVRKNFLAALESAPLGTTP------------EKVQNGKDFYKFLFTSNPDVRKY----------------FKGA-------------ESFTADDVQKS
OOP03092_2 : -------------KVVPLGKTP------------AEIQHGTDFYKYMFGHHPDLRKY----------------FKGA-------------ENFTPDDVQKS
OOP00214_1 : --LISNSRPLASXSVVPLGKTP------------AEIQNGTDFYKYLFGHHQDLRKY----------------FKGA-------------ENFTPDDVQKS
PTP03438_1 : MSVEGKKLLKESMAKVMVGNDA------------TGQQNGKDFYKFFFTNFADLRKY----------------FKGA-------------EHFTADDVQGS
OVP00634_1 : GTKAKCLKVMNESGRVGNCD--------------AAKQDGLNFYKYMFGHHPDLRVY----------------FKGA-------------ENFTPTDVQNS
OVP04040_1 : SHSGTKAKCLKVMNESGRVGNCD-----------AAKQDGLNFYKYMFGHHPDLRVY----------------FKGA-------------ENFTPTDVQNS
SSP04654_1 : NCKGIVAIVEKVFDKLELKDKS------------VKEVFYNTAFVDSVYEYAGRRRP----------------SCPS-----------INEHRPKCTAHSH
SSP00231_1 : KLKKLPFISLEVLRLGGEHGAIPHG--KDFYKLXVIVKICIYILXSMFGNYPQLRVY----------------FKGA-------------ENYTPEQVGAS
SSP02226_1 : DSTEVKKVARKSLEVLRLDDEH------------GAIPHGKDFYKFMFGNYPQLRVY----------------FKGA-------------ENYTPEQVGAS
Trichostrongylu : ---EIRKDALSALDVVPLGSTP------------EKLENGREFYKYFFTNHQDLRKY----------------FKGA-------------ETFTADDIAKS
SSP04285_1 : -RNSTKEFLTSHQRALLIRSWNKSQKT-------GLDNIGAMIFFKIYVNDQSVGKM----------------FGL--------------QGIPLSELKYK
Toxocara : ----MATACLKSLESAQCGTCD------------KAIENGTGFYALLFDKHPELRHY----------------FKGN-------------ENLTGAEVKKS
TCP00537_1 : SAESPRELCMKSLKDVHVGTGD------------HAKQCGIDLYKHMFEHYPDLRKF----------------FKNR-------------ENYTAEDVQKD
TDP00173_1 : -MADVKKACLESLSVVPLGRGP------------AEVQNGKDIYKYLFGHHPDLRKY----------------FKGA-------------ENFTPDDVQKS
TDP00009_2 : ---DVKKACLESLKVIPLGRTP------------AETQHGTDFYKYLFGHHPDLRKY----------------FKGA-------------ENFTPDDVQKS
TDP00009_1 : -MADVKKACLESMKVIPLGKTP------------AETQHGTDFYKYLFGHHPDLRKY----------------FKGA-------------ENFTPDDVQKS
TDP00127_1 : -MADVKKACLASLGVVPLGKTP------------AETQHGIDFYKYLFGHHPDLRKY----------------FKGA-------------ENFTPDDVQKS
TDP00084_1 : -MADVKKHCMESLGTVPLGRAP------------EQVQNGKDFYKYLFTNHPDLRKY----------------FKGA-------------ENFTADDVQKS
TDP00032_1 : -MADVKKACMESLKVVPLGRTP------------AEIQNGTDFYKYLFGHHPDLRKY----------------FKGA-------------ESFTPDDVQKS
TDP00008_1 : GPEDVRKNSLAALESVALGTTP------------EKVQNGKDFYKYLFTEHPEVRKY----------------FKGA-------------ESFTADDVQKS
TDP00008_4 : GPEDVRKNSLAALESVALGTTP------------EKVQNGKDFYKYLFTEHPEVRKY----------------FKGA-------------ESFTADDVQKS
TDP00008_2 : GPEDVRKNSLAALESVALGTTP------------EKVQNGKDFYKYLFTEHPEVRKY----------------FKGA-------------ESFTADDVQKS
TDP00008_3 : GPEDVRKNSLAALESVALGTTP------------EKVQNGKDFYKYLFTEHPEVRKY----------------FKGA-------------ESFTADDVQKS
TDP01113_2 : SDADVKKNFLAALESAPLGSTP------------EKIQNGKDFYKFLFTNNPEVRKY----------------FKGA-------------ESFTADDVQKS
TDP01113_1 : SDADVKKNFLAALESAPLGSTP------------EKIQNGKDFYKFLFTNNPEVRKY----------------FKGA-------------ESFTADDVQKS
TMP01615_1 : QVFTNMTILKDQLNQVPP-----------------NPTNGALFYKTFFTVVPPEYSK----------------LFGM-------------DKINPADVPGM
TVP00688_1 : TYASSASIVKEQVAQIEV-----------------NEENGAKLYEIFFTVKPEFHKF----------------FQMQ-------------HAPEGKDILHN
TMP00180_1 : TYASSASIVKDQVSRIEV-----------------NEENGGKLYETFFTVKPEFHKF----------------FDLQ-------------HAPEGKDIVHN
TVP00077_1 : ------TILKDQLNQVPVN-----------------ATNGGLFYKTFFTVAPPDYSK----------------FFGM-------------DKINPADVPGM
XIP00721_1 : MASQHQATLKKQMAKVQV-----------------NPQNATAFYSHFFTVAPDLRVF----------------FKGA-------------ETMDPKDVPAS
ZPP00218_1 : XKDLXKKSLEKALIGIDED----------------EVQNGLNFYKFFFTNFPDLRVY----------------FKGA-------------EKFTADDVQKS
MIP00306_1 : --MVNGELLKKQIQQFKL-----------------GKDAAADYYKELFSKYSDVADA----------------YGGV-------------------DPETV
RSP00034_1 : SMGVNTELLQKQVQALKL-----------------SKETGGDYYKELFAQHSDVCDA----------------YGGI-------------------DPDAV
OVP06929_1 : LTLSQRNIVKGCMDKAKD-------------------DIAERIYRRIAEKREDFRK-----------------FVEA---------------------LSE
GRP00115_1 : FTPKAQKNVKMVNSELLQKHVQQL---------KMGKPAAIDYYKELFSKHSDVAEA----------------YGGI-------------------EPDAV
MAP00909_1 : LTPQQVSNLRRSWKHINTK---------------GLYDVIRRAFSKLESSAPNVRSA----------------FKNN-------YLDNQQQKNIGGTSNNK
HGP00385_1 : REEIQAVGVQSLKQLKIGAGE-------------AEFPKGKAFYKCVFLNYPTPRVY----------------LXGA-------------EKLEAEDVQKL


helices : eeEeeEEeeEEeeEeeEEe-----------------------------------FffFfffFFfggg------------------G----------ggggG
 : --|--||--||--|--||------------------------------------|--|---||----------------------|--------------|
ZK637.13 : ERFDKQGQRILLACHLLANVY------------------------TNEEVFKGYVRETINRHRIYK-------------------M----------DPALW
CBG0687 : ERFDKQGQRILLACHLIANVF------------------------TNEEVFKAYVRETINRHRIYK-------------------M----------DPALW
CRZK63 : ERFDKQGQRILLACHLIANVY------------------------TNEEVFKAYVRETINRHRIYK-------------------M----------DPALW
F49E2.4 : KEFHLQAHRIQNFLDTAVGSL----------------------GFCPISSVFDMAHRIGQIHFYRG-------------------V--------NFGADNW
CBG16082 : KEFHLQAHRIQNFLDTAVGSL----------------------GFCPISSVYDMAHRIGQIHFYRG-------------------V--------NFGADNW
CRF49 : KEFHLQAHRIQNFLDTAVGSL----------------------GFCPISSVYDMAHRIGQIHFYRG-------------------V--------NFGADNW
C52A11.2 : PKFGGHILRFYEFMEQLTSML---------------------GTSENLTGAWQLVRKTGRSHVRQGFLEQNQNQ-----------M------EKNYFEIVI
CBG03023 : PKFGGHILRFYEFMEQLTSML---------------------GTSENLTGAWQMVRKTGRSHVKQGFLEQNQNQ-----------M------EKNYFEVVI
CRC52 : PKFGGHILRFYEFMEQLTSML---------------------GTSENLTGAWQMVRKTGRSHVKQGFLEQNQNQ-----------M------EKNYFEVVI
C28F5.2 : ATMEVHIKLTTKFFDELLVSL------------------------DDETEFVNKIRGIGSAHAILAKGSN---------------F----------SSDIW
CBG13047 : ATLEVHIKLTTKFFDELLATL------------------------DDENEFVAKIRGIGSAHAILAKGSN---------------F----------SSDIW
CRC28 : ATMEVHIKLTTKFFDELLSTL------------------------DDENEFVAKIRGIGSAHAILAKGSN---------------F----------SSDIW
W01C9.5 : RTVADHAKYLLQLLDKIIEGD----------------------------VDSEFLREIGANHVCLKHESG---------------F----------STQEW
CBG00571 : RTVADHAKYLLQLLDKIIEGD----------------------------VDSEFLREIGANHVGLQRDTG---------------F----------SSLEW
CRW01 : RTVADHAKYLLQLLDKIIEGD----------------------------VDSEFLREIGANHVNLKHENG---------------F----------STQEW
C29F5.7 : LSSTIQAFFYKLIITYELNDD----------------------------QVREACEQLGARHVDF--------------------I------SRGFNSHFW
CBG02622 : LSSTIQAFFYKLIITYELNDD----------------------------QVREACEQLGARHVDF--------------------I------SRGFNSHFW
CRC29 : LSSTIQAFFYKLIITYELNDD----------------------------QVRDACEQLGARHVDF--------------------I------SRGFNSHFW
F19H6.2 : PRFRQHALVYTKTLDFVIRNL------------------------DYPGKLEVYFENLGKRHVAMQGRG----------------F----------EPGYW
CBG00138 : PRFRQHALVYTKTLDFVIRNL------------------------DYPGKLEVYFENLGKRHVAMQGRG----------------F----------EPGYW
CRF19H6.2 : PRFRQHALVYTKTLDFVIRNL------------------------DYPGKLEVYFENLGKRHVAMQGRG----------------F----------EPGYW
R01E6.6 : NNRLKQLVEDIVAHIHDADFI------------------------------ESVSKQYGEEHVELKQYG----------------F-------KPDFWVAV
CBG07422 : NNRLKILVEDIVAHIHDSDYI------------------------------ELVSKQYGEEHVELKQYG----------------F-------KPDFWVAV
CRR01 : NNRLKQLVEDIVAHIHDADFI------------------------------ESVSKQYGEEHVELKQYG----------------F-------KPDFWVAV
F46C8.7 : STLRDHSKNCVKMIDSVIKNF------------------DVEKSKRTDTSSENDPRVIGRAHSILKPYG----------------L----------AGNYW
CBG16720 : STLRDHSKNCVKMVDSVIKNF------------------DVEKSKRSDTGTENDPRVIGRAHSILKPYG----------------L----------AGNYW
CRF46 : STLRDHSKNCVKMVDSVIKNF------------------DIEKSKRTDTGTENDPRVIGRAHSILKPYG----------------L----------AGNYW
R13A1.8 : HPVRRHARLFTSILHISVKNV-----------------------DELEAQVAPTVFKYGERHYRPD-------------------I------TPHMTEENV
CBG05809 : HPVRRHARLFTNILHISVKNV-----------------------DELEAQVAPTVFKYGERHYRPD-------------------I------TPHMTEENV
CRR13 : HPVRRHARLFTNILHISVKNV-----------------------DELEAQVAPTVFKYGERHYRPD-------------------I------TPHMTEENV
C06H2.5 : CDLNSHTKLLCELLDSLMTDL-----------------------HQPAKIVLAKCQDVGAAHVNMNEKCCG--------------V-------------VF
CBG23115 : CDLNSHAKLLSELLDSLMTDL-----------------------QQPAKIVLAKCQDVGAAHVNMNEKCCG--------------V-------------VF
CRC06H2.5 : CDLNSHAKLLCELLDTLMTDL-----------------------HQPAKIVLAKCQDVGAAHVNMNEKCCG--------------V-------------VF
C26C6.7 : NFIKAHCKAVAELIDQVVENL------------------------DHLDNVTGELMRIGRVHAKVLRGE----------------L----------TGKLW
CBG11881 : NFIKAHCKAVAELIDQVVENL------------------------DHLDNVTGELMRIGRVHAKVLRGE----------------L----------TGKLW
CRC26C6.7 : NFIKAHCKAVAELIDQVVENL------------------------DHLDNVTNELMRIGRVHAKVLRGE----------------L----------TGKLW
F52A8.4 : KEFRSQALKFVQTLAQVVKNI------------------------YHMERTESFLYMVGQKHVKFADRG----------------F----------KHEYW
CBG11915 : KEFRSQALKFVQTLAQVVKNI------------------------YHMERTESFLYTVGQKHVKFADRG----------------F----------KHEYW
CRF52 : KEFRSQALKFVQTLAQVVKNI------------------------YHMERTESFLYMVGQKHCKFADRG----------------F----------KHEYW
T22C1.2 : STLDYHNLQIVEFLQKVMQSL------------------------DEPDKISKLCQEIGQKHAKYRRSKGMK-------------I------------DYW
CBG08252 : STLDYHNIQIVEFLQKVMQSL------------------------DEPDKISKMCQEIGQLHAKYRRSKGMK-------------I------------DYW
CRT22 : STLDYHNIQIVEFLQKVMQML------------------------DEPDKISKLCQEVGQKHAKYRRSKGMK-------------I------------DYW
C18C4.1 : DFYFRHHCASLQAALNMIIQN-----------------------KDDKSGMRRMLNEMGAHHFFYDACEPH--------------F------------EVF
CBG09371 : DFYFRHHCASLQAALNMIIQN-----------------------KDDRNGMRRMLNEMGAHHFFYDACEPH--------------F------------EVF
CRC18C4.1 : DFYFRHHCASLQAALNMIIQN-----------------------KDDRNGMRRMLNEMGAHHFFYDACEPH--------------F------------EVF
R11H6.3 : ATLRDHTHFFVSLVSQVVSSL-----------------------EKEPAKILEHLDHIGQSHAYLKRYG----------------F----------KSSHW
CBG04577 : ATLRDHTHFFVSLVSQVVSSL-----------------------EQEPAKILDHLDHIGQSHAYLKRYG----------------F----------KSSHW
CRR11 : ATLRDHTHFFVSLVSQVVSSL-----------------------EQEPAKILEHLDHIGQSHAYLKRYG----------------F----------KSSHW
C23H5.2 : KEIEFHALRFMQVLESVVKTL------------------------DNPETLNPLCDNLGRVHGRLSESRG---------------F----------RTHHW
CBG10551 : KEIEFHGLRFMQVLESVVKTL------------------------DNPESLNPLCDNLGRVHGRLSESRG---------------F----------RTHHW
CRC23 : KEIEFHALRFMQVLESVVKTL------------------------DNPESLNPLCDNLGRVHGRLSESRG---------------F----------RTHHW
CBG24799 : PGFEAVAAQYLKVFDDVITAV--------------------EEKPGDVQTACDRLVAVGKMHRQK--------------------V-------SGMNVSMF
CRT06 : PGFESMALQYLKIFDDVITAV--------------------EEKPGDVQTACDRLSAVGKMHRQK--------------------V-------SGMTATQF
T06A1.3 : PGFEAIAAQYLKVFDDVITAV--------------------EEKPGDVQTACDRLQAVGKMHRQK--------------------V-------SGMDGTMF
Y17G7B.6 : KTEYDHCKCMVGIFERLIENL------------------------ENINEQLTMIRHYGEKHAQMAESG----------------F----------TGAMI
CBG21021 : KTEHDHCKCMVGIFERLIDNL------------------------DNINEQLNMIRHYGEKHAQMAESG----------------F----------TGAMI
CRY17 : KTEHDHCKCMVGIFERLIENL------------------------DNINEQLTMIRHYGEKHAQMAESG----------------F----------TGAMI
Y57G7A.9 : ADRISQFFHQLVEDDVLMDTV----------------------------ELKKACYDLGRQHSSYSKQQ----------------F----------KMSYW
CBG07112 : ADRISQFFHQLIEDDVLMNTV----------------------------ELKKSCYDLGRQHAAYSKKQ----------------F----------KISFW
CRY57 : ADRISQFFHQLIEDDVLLNTV----------------------------ELKKCCYDLGRQHSAYSKKQ----------------F----------KISFW
Y15E3A.2 : ETENRHVKYFVDLVQSCVDNL-----------------------ENLETGVKPWLDLIGRGHANFK-------------------I----------TGKHW
CBG07681 : ETEQRHIKYIVELVQSCVGKL-----------------------DNLEEGLKPLVELLGRGHSNFR-------------------I----------TGPHW
CRY15 : ETENRHIKYFVDLVQSSVDNL-----------------------EDLEASVKPWLDLIGRGHSDFK-------------------I----------TGKHW
Y58A7A.6 : DYFKSHASNLALVLNLVVTNL-----------------------QDNFDQAQDALQALGYQHLHL--------------------I----------DRTHF
CBG08670 : DYFKSHAANLALVLNLVVTNL-----------------------QDNFEQAQDALQALGYQHLHL--------------------I----------DRTHF
CRY58 : EYFKSHASNLALVLNLVVTNL-----------------------QDNFEQAQDALQALGYQHLHL--------------------I----------DRTHF
C18C4.9 : ERLKKHAQLFTVLMDDLIANL------------------------DSPSATVAGLREAGEKHVWPTRNQ----------------Y------GCPFHAHLL
CBG09371 : RLKAEHAQLFTVLMDDLITNL------------------------DSPSATVAGLREAGEKHVWHVKNQ----------------Y------GCPFHAHLM
CRC18C4.9 : ERLKRHAQLFTVLMDDLIASL------------------------DSPSATVAGLREAGEKHVWPVKNQ----------------Y------GCPFHAHLM
R90.5 : DCSVMHCRVTVEILDTVIKNL-----------------------DNDHARITQYLTEIGKQHRHLKAEG----------------L----------SSAVW
CBG09511 : DCSMMHCRVTVEVLDTVIKNL-----------------------DADHSRVTQYLTEVGKQHRHLKAEG----------------L----------SSAVW
CRR90.5 : DCSMMHCRVTVEVLDTVIKSL-----------------------DTDHYRVTSYLTEVGKQHRHLKAEG----------------L----------SSAVW
C36E8.2 : NLSKGQRYENSQYLKQFLLGI--------------------VENIMDIDEINRISEEFGCNHVQFRANG----------------F---------KPDFFA
CBG03635 : KLTKKQRYENSSYLKQFLLGV--------------------VDNLTDIDEINRLAEDFGCNHVQFRANG----------------F---------KPDFFA
CRC36 : NLSKAQRYENSNYLKQFLLGI--------------------VESLTDIDEINRISEEYGCNHVQFRLYG----------------F---------KPDFFA
Y75B7AL.1 : AKFQEIGGRITSFISELLELM---------------------QNNQPESYIVMRIRRVGAVHYDKG-------------------I--------VFTSSVW
CBG06424 : AKFQEIGGRISGFIAELLELM---------------------RTNQAESYIIMRIRRVGAVHYDKG-------------------I--------VFSSSVW
CRY75 : AKFQEIGGRISGFITELLNLM---------------------QSNQAESYIIMRIRRVGAVHFDKQ-------------------I--------VFSSSAW
F35B12.8 : QKFRTHVGKFQRFITGIIDML--------------------SKGVESSDQIVEILRIVGRQHGNVRTMS----------------F----------TAEKW
CBG23302 : QKFRTHVGKFQRFITGIIDML--------------------SKGVESSDQIVEILRIVGRQHGNVRTMS----------------F----------TAEKW
CRF35B12.8 : QKFRTHVGKFQRFITGIIDML--------------------SKGVESSDQIVEILRIVGRQHGNVRTMS----------------F----------TAEKW
F21A3.6 : VELRRHASVYLNGLGKIIDSM------------------------RDEEALGKSMSRIAVAHIKWN-------------------V----------QRNHV
CBG18593 : VELRRHASVYLKGLGKIIESM------------------------RDEEELRKSMSRIALAHIKWN-------------------V----------QRNHV
CRF21A3.6 : VELRRHASVYLKGLGRIIESM------------------------RDEEELRKSMSRIALAHIKWN-------------------V----------QRNHV
C06E4.7 : KRARSVADGVNCFFNKVISKL---------------------MDANYVEEIQDLSLQLGAMHFRMK-------------------V--------WFQAENW
CBG05824 : SRARSVADGVNCFFNKVISKL---------------------MDHNYIEEIQDLSLQLGAMHFRMK-------------------V--------WFQAENW
CRC06E4.7 : KRARSVADGVNCFFNKVISKL---------------------MDTNYVEEIQDLSLQLGAMHFRMK-------------------V--------WFQAENW
F56C4.3 : NEFAFQAMRFMQVIEGAVKAL------------------------DHLTSLDVILDNLGRRHGKLEVNGK---------------F----------RSYYW
CBG04428 : NEFAFQAMRFMQVIEGAVKAL------------------------DHLTSLDVILDNLGRRHGKLEVNGK---------------F----------RSYYW
CRF56 : NEFAFQAMRFMQVIEGAVKAL------------------------DHLTSLDVILDNLGRRHGKLEVNGK---------------F----------RSYYW
R102.9 : REFQLQAHRIQNFLDTAVSSL--------------------------------GFCPIGNIHQMAYRIGQ---------------I------------HFY
CBG17640 : REFQLQSHRIQNFLDTAVSSL--------------------------------GFCPIGNIHQMAYRIGQ---------------I------------HFY
CRR102 : REFMLQSHRIQNFLDTAVSSL--------------------------------GFCPIGNIHQMAYRIGQ---------------I------------HFY
C09H10.8 : RDFLNILIKVHKCDPEMIKTL-------------------------------SFRLGARHRHYMNEGNDNC--------------F----------WAPFA
CBG02965 : RDFLNILIKVHKCDPEMIKTL-------------------------------SFRLGARHRHYMNEGNDNC--------------Y----------WAPFA
CRC09 : RDFLNILIKVHKCDPEMIKTL-------------------------------SFRLGARHRHYMNEGNDNC--------------Y----------WAPFA
Y22D7AR.5 : NEFIFHAVRFLQVIESTMTHL------------------------EDPAQLDAVFLNLGKIHAKHEEQ-----------------L--------GFSAHYW
CB_Y22 : SNEFIFHAVRFLQVIESTMSH-----------------------LDDPSQLDAVFLNLGKIHAKHEEQ-----------------L--------GFSAHYW
CR_Y22 : NEFIFHAVRFLQVIESTMTHL------------------------DDPSQLDAVFLNLGKIHAKHEEQ-----------------L--------GFSAHYW
Pp27.56 : FYFRHHCASVQAAITMIMENK------------------------DDIVSLTRVLNEVGAHHFFYDAY-----------------------------EPHL
Pp42.55 : DCTAMHSRVTIELLDTAIRNL-----------------------DADHAKLTAYLIEVGRSHRPLRQEG----------------L----------AIAVW
Pp10.104 : EFVKAHCKAVAELVDQVVENL------------------------DNLDNMNEELMRIGRVHAKMTRGG----------------V----------TGKLW
Pp94.7 : AKYQEIANRITSFIEELLKMM---------------------LDGAPDTCLQMRIRRAGAIHHSRNIK-----------------I----------SSTVW
Pp50.31 : LSSFCTHSRFSNRRKSMMQCI------------------------DSPDGLEPLFRNLGQIHGRHQEQLG---------------F----------RPHYW
Pp106.6 : QAFKKHAANFALVLDLVIKNI-----------------------PDNVDSCCQALQALGGQHVSLRDRG----------------F----------DSIYW
Pp68.42 : NKIKAVHHAIPKSLQILVQQL-------------------------DETQMRITCEALGARHIDFVSRG----------------F----------NSAFW
Pp14.29 : KEFHLQAHRIQNFLDTAVGSL----------------------GYCPVTSIYDMAHRIGQIHFYRGVN-----------------F----------GADNW
Pp84.53 : ASLRDHAKITVKMLDLIIRNL------------------DTEKAKRTDTGSNLDPRLVGRAHGPLRPYG----------------L----------TGIFW
Pp68.66 : VSFSNLYTLIEDFFTKVIVTQ-----------------------HLDDDLTVDCAQALGARHIDFVGQG----------------F----------NSAFW
Pp168.42 : TEFKDQALKFVQVIDTVVWGI------------------------IDGDKSEPFLYDVGQRHVQYASRG----------------F----------KASYW
Pp326.1 : NEFRIQALRLVQTIAKVLDTV------------------------DDLVKLEALLYKVGHRHVHYLPAG----------------L----------DSVYW
Pp2.166 : SEFRIQALRLVQTIEKALDKV------------------------DNIEKLERYLYALGHRHVHYLPVW----------------L----------DPEYW
Pp2.164 : TEFRTQALRLVTTIAKVVENL------------------------PHLKGLDMHLYKLGHRHVKYLSNA----------------L----------KPLYW
Pp82.11 : SEFRIQALQLVQAMAKTLDKI------------------------EDATALELYLYQLGQRHIHYLPPR----------------I----------DVKTW
Pp360.2 : EEKQQYVDSLKEFLTLVCTSL------------------------MDSEFVQKTGFEYGQKHANLRTKG----------------F----------KPDFF
Pp14.30 : ERFEKQGQRILLAVHVLAETY------------------------ANQEVFKAYVRETINRHRIYK-------------------M----------DPALW
Pp38.62 : PRFRQHALVYTKTFDYVVKNL------------------------EFPEKLEHHFDLLGRRHVQYQGRG----------------F----------DPSFW
Pp5.100 : EMLKQHSLVYTKTIDYVVKNL------------------------EFPHYLEQHFELLGRRHVQYQGRG----------------F----------DPSWW
Pp277_1 : PKLGGHVARFTDLFEQITSML---------------------GVSENLTGAWQLIRKTGRVHAKQAFLEQNFNQ-----------L----------EKNYF
Pp43.86 : ATLDEHIKLAAKFFDDLLAVF------------------------DDEEEFRTLIRRMGAVHAV-LARSCN--------------F----------GGEIW
Pp94.50 : CDLAAHLKYLTELFDALFADL-----------------------NQPAKQAETRCYEIGVAHYNMTEK-----------------C----------AGNIW
Pp137.9 : DGLKNHASVYMAGLQHIVESL------------------------DDDATLGEAIQRIAMSHAKWK-------------------I----------KKYHL
Pp38.51 : TVKLKELVEEVVVRIDDVETI------------------------------ASMCRNYGEEHVELKSAG----------------F----------KPDFW
Bm1_37235 : RQFQPSSAAPLQVLESGINSL------------------------DDLKAFDPILDNLGRRHGKLESSSG---------------F----------RLYYW
Bm1_31825 : SAELRDYLNNVVSKVHNVAAV------------------------------EEMSRHYGERHVPLKKYG----------------F----------KPDFW
Bm1_41355 : SVLRNFAHTXMNALKKIVESL------------------------NNEQMPYEVLQRISAKHARHN-------------------I----------QMHHM
Bm1_13755 : EQRCELANSLRSYLKNVVNQL------------------------MDRAAVQRISEDFGARHVQYRSSG----------------F----------RPDFF
Bm1_27145 : EQSTELVMALKKFLTDVVQNI----------------------TNSEKIRQISSKYGIEQAHKRSWG------------------F----------KADFF
Bm1_17795 : YDVKEHADSLIELIDFALREI-----------------------HSSIKVVQDRCMLMGAAHCNTCENS----------------M-----------SSSW
Bm1_02240 : SFNKAHAKAVGEMLDKIVDNL------------------------ENLESMSGYLFSIGVTHANLARRQ----------------I----------SKEIW
Bm1_33065 : ATPRDHAKETVKLIDYAIKNL------------------EVNDKERNDTGSDYDPFLLGKAHGRLRPYG----------------F----------TGNYW
Bm1_21435 : PRFRRHAIVFTKSFDYIVKNV------------------------AYKEKLEQHFQALGERHTTLQGRG----------------F----------DPGYW
Bm1_50430 : ILYFLNRQKLLLAVRIIINTY------------------------DDPETFRAYARETINRHIKFK-------------------I----------DRALW
Bm1_46940 : PKLGGHIVRFTDCLXQLTNMI---------------------GYTENLIGAWQLARKTGRAHSKQSFLE----------------M------NQNEEKNYF
Bm1_24705 : GTLEEHVKLMIEFFDDLVNNA------------------------QDEENMSNKIRKVGQCHAILTQCS----------------F----------SADIW
Bm1_04635 : HSIFRQMRLFTNLIALAVRHD-----------------------NELETEIAPAVFRYGQRHYKFAAEY----------------F----------NEGTV
Gossypium : TKLKPHAMSVFVMTCESAVQL---------------------RKAGKVTVRESNLKKLGATHFKYG-------------------V----------VDEHF
Malus : PKLKPHAMSVFVMTCESAVQL---------------------RKAGKVTVRESTLKRLGGVHFKSG-------------------V----------VDEHY
Glycine : PKLKPHAVSVFVMTCDSAVQL---------------------RKAGKVTVRESNLKKLGATHFRTG-------------------V----------ANEHF
Alnus : PKLKSHAMSVFLMTCESAVQL---------------------RKAGKVTVRESSLKKLGAVHFKHG-------------------V----------VDEHY
Citrus : PKLKPHATTVFVMTCESAVQL---------------------RKAGKVTVRESNLKDLGATHFKYG-------------------V----------ADEHF
Nemvec141000032 : NALHGHAKRVMKAVENAVMSID------------------------DVMSFSAYLEELGRRHKTRA-------------------L----------KPSYL
Nemvec3000224 : RSLYLHAKRVMVAVENAVTVLD------------------------DAETFESYLINLGGRHLPWG-------------------V----------TKDHF
Nemvec50000067 : PALYGHARRVMKSVDNAVASIE------------------------NVQVYSAYLYELGTRHQTRQ-------------------L----------SEEQL
Nemvec7000121 : ERFLFQSRKFMEMINSAVDRLN------------------------DISLLVMILKSLGEVHWTKFK------------------I----------KPEYY
Nemvec76000030 : RSLQGHTKRVMKVVENAVNSLE------------------------DGHALMEYLQELGRRHKTRQ-------------------I--------KPTVSNL
Paragonimus1 : EGIKYYARTLVEDLVKMLTAAAK------------------------DDELQKVLVHSGHQHTTRK-------------------V----------TKQQF
Paragonimus2 : EGIKYYGRTLATELKLLVANAAN------------------------ASELEAVVVKNSKDHTTRN-------------------V----------TSAQF
Clonorchis : EGIKYYGQTFADSILEMLQCASD------------------------DGKLEAVLEKSGKEHITRN-------------------V----------TKQQF
Isoparorchis : DGAKYYAKTLINDLVELLKASTD-----------------------EATLNTAIARTATKDHKPRN-------------------V----------SGAEF
Paramphistomum : DGIKHYARTLTEAIVHMLKEISN------------------------DAEVKKIAAQYGKDHTSRK-------------------V----------TKDEF
Schistosoma : EGIKYYARTFVAAFVPMIQAAAN------------------------KCELDKLCLEEAILHRTRP-------------------V----------DEKIF
Pseadoterranova : AFFVKQGHKILLALRMLCSSYDDEP------------------------TFDYFVDALMDRHIKDDIH-----------------L----------PQEQW
Heterorhabditis : ERFEKQGQRILLAMHILVNIINQ------------------------MPVFRAYARETVNRHRQFK-------------------M----------EPALW
Mermis_Eye : PRFLRQGQRVLLSMELMIELADKPQ------------------------LFDAYVRDLLDKHKQIKG------------------I----------DYDLY
Mermis_Body : ARFLRQGQRLLLSMELMIELADKLQ------------------------LFEPYVREMLDKHKRFKG------------------I----------DYDLY
Toxoperient : EFFAKQGQKILLACHVLCATVDDME------------------------TFNAYSHEVLDRHERDHVE-----------------L----------PASAW
GRP00348_1 : ERWQQMTGKLKEFLELVVRKVDNIEM------------------------VERLSRKYGEEHVELKCFG----------------F----------KPDFW
ACP03829_1 : DRFEKLGTSILLSVHILANTYDN------------------------EPVFRAFCRDTIDRHVNRG-------------------L----------DPVLW
ACP01487_2 : ERFDKQGQRILLAVYILADTFDD------------------------EPTFRAYARETVNRHRQFK-------------------M----------DPELW
ACP00369_1 : CEFSFQALRFVQVIEGAVMSLDNL------------------------PALDPILDNLGRRHGKLGVNGK---------------F----------RTYYW
ALP00043_1 : DHFKKQGQRLLLACHVLAHLEND------------------------PPSFKAYAREIIDRHLRMSVH-----------------L----------EPKLW
AscarisMb : DHFKKQGQRLLLACHVLAHLENDPA------------------------SFKAYAREIVDPHLRMSVH-----------------L----------EPKLW
AscarisHb : PFFAKQGQKILLACHVLCATYDDRE------------------------TFNAYTRELLDRHARDHVH-----------------M----------PPEVW
Syng_Intracell : ERFEKQGQRILLAVYILADTFDDEQ------------------------TFRAYARETVNRHRQFK-------------------M----------DPSLW
Syng_Cuticul : EKFRTLGNAFILAIHIVANTYDN------------------------EPVFRAYVRDNIARHVERG-------------------L----------EPSLW
ASP00019_1 : PFFAKQGQKILLACHVLCATYDDRE------------------------TFNAYTRELLDRHARDHVH-----------------M----------PPEVW
ASP00780_1 : DRFAKQGQRILLACHILANTYDD------------------------PDTFKAYARETVNRHR-QFK------------------M----------EPSLW
ASP17423_1 : EHFKKQGQTLLLACHVLAHLEND------------------------PSSFNAYCREIIDRHLRANVH-----------------L----------DPKLW
AYP02881_1 : ATMDVHIRLMVKFFDDLLATLDD------------------------EAECTKRMKQIGTSHAVLARTCG---------------F----------TSDIW
AYP03578_1 : DRFEKQGNALLLSVHVLANTYDN------------------------EEVFRAFCRDTINRHATRG-------------------L----------DPVLW
AYP00272_1 : DRFEKLGTSILLSVHILANTYDN------------------------DAVFRAFCRDTIDRHVNRG-------------------L----------DPALW
AYP01816_1 : ERFDKLGHAILLHVHVLANTFDN------------------------EPVFRAFTGEVINKHVTRG-------------------L----------DPALW
AYP00544_1 : ERFDKLGHAILLHVHVLANTFDN------------------------EPVFRAFTGEVISRHVTRG-------------------L----------EPVLW
DIP00455_1 : PRFRRHAIVFTKSFDYIVKNVA------------------------YREKLEQHFQALGERHTAFQGRG----------------F----------DPGYW
HCP00759_3 : ERFAKQGTALVMTVHIFANLYDN------------------------DMVFRGFCRDLMNRHVERK-------------------I----------DPALW
HCP00759_2 : ERFAKQGTALVMTVHIFANLYDN------------------------DMVFRGFCRDLMNRHVERK-------------------I----------DPALW
HCP00759_1 : ERFAKQGTALVMSVHIFANLYDN------------------------DMVFRGFCRDLMNRHVERK-------------------I----------DPALW
HCP00786_1 : DRFAKQGTALLMTVHIFANTYDN------------------------DMVFRSLCRDLMDRHVGRN-------------------V----------DPSLW
HCP13111_1 : ERFKKQGTRLLTAVHVLANTYDN------------------------DAVFRGFVRDLIHRHSDKR-------------------I----------DPKEW
HCP00770_1 : DRFAVQGMALLTSVHILADTYDN------------------------EMIFRAFVRDLMNRHKERG-------------------L----------DPKLW
HCP00770_3 : DRFAVQGMALLTSVHILADTYDN------------------------EMIFRAFVRDLMNRHKERG-------------------L----------DPKLW
HCP00208_1 : ERFKKQGTRLLTAVHVLANTYDN------------------------DAVFRGFVRDLIHRHSDKR-------------------I----------DPKEW
HCP00202_4 : DRFVKQGGNLLTGVHVLANTYDN------------------------DMVFRAYVRDLMNRHTSKE-------------------I----------DPKQW
HCP00208_2 : ERFKKQGTRLLTAVHVLANTYDN------------------------DAVFRGFVRDLIHRHSDKR-------------------I----------DPKEW
HCP01314_3 : DRFVKQGANLLTGVHVLANTYDN------------------------DMVFRAYVRDLMNRHTTKE-------------------I----------DPKQW
HCP00202_3 : DRFVKQGGNLLTGVHVLANTFDN------------------------DMVFRAYVRDLMNRHTSKE-------------------I----------DPKQW
HCP00333_2 : DRFVKQGGNLLTGVHVLANTFDN------------------------DMVFRAYVRDLMNRHTSKE-------------------I----------DPKQW
HCP00202_2 : DRFVKQGANLLTGVHVLANTYDN------------------------DMVFRAYVRDLMNRHTTKE-------------------I----------DPKQW
HCP00202_1 : DRFVKQGGNLLTGVHVLANTYDN------------------------DMVFRAYVRDLMNRHTAKE-------------------I----------DPKQW
HCP00770_2 : DRFAVQGMALLTSVHILADTYDN------------------------EMIFRAFVRDLMNRHKERG-------------------L----------DPKLW
HCP00202_5 : DRFVKQGGNLLTGVHVLANTFDN------------------------DMVFRAYVRDLMNRHTAKE-------------------I----------DPKQW
HCP02815_1 : DRFATQGSALLTSVHILADTYDN------------------------EMIFRAFVRDLMNRHKERG-------------------L----------DPKLW
HCP08501_1 : DRLQKIGSALITTVHVFVNIYDNPX----------------------EMVFRAFCRNLMDKHVGRG-------------------V----------DPSLW
HGP06223_2 : ERFEKQGQRILVAMHLIANVYSN------------------------EMLFNAYVRETVNRHRQFK-------------------M----------EPSLW
MJP04640_1 : ERFEKQGQRILLAMHLTARVYAD------------------------EMVFESYIQETINRHRQFK-------------------M----------EPALW
MIP00586_2 : ERFEKQGQRILLAMHLTARVYAD------------------------EMVFDSYIQETINRHRQFK-------------------M----------EPALW
MHP02564_2 : ERFEKQGQRILLALHLTARVYSD------------------------EMVFDSYIQETINRHRQFK-------------------L----------EPGLW
NAP00088_1 : ERFEKQGNALLLSVHILANTYDN------------------------EEVFRAYCRDHTNRHAAKG-------------------I----------DPHYW
Nippo_Body : DRFEKLGSGLLLSVHILANTFDN------------------------EDVFRAFCRETIDRHVGRG-------------------L----------DPALW
Nippo_cuticul : KRFEKQGTALLLAVHVLANVYDN------------------------QAVFHGFVRELMNRHEKRG-------------------V----------DPKLW
NBP00095_1 : DRFLKLGNGLLLSVHILANTMDN------------------------EDVFRAFCRDTIDRHVGRG-------------------L----------DPSLW
NBP00124_1 : DRFEKLGTGLLLSVHVLANTYDN------------------------EDVFRAFVRDTIDRHVSRG-------------------L----------DPALW
NBP00197_1 : KRFEKLGTSLLLAVHILANTYDN------------------------QEVFHGWVRELMNRHEKRG-------------------V----------DPKLW
NBP00328_1 : ERFEKLGTSLLLAVHILANTYDN------------------------EAVFDGWVRELMNRHEKRG-------------------V----------DAKLW
OOP00190_2 : DRFAVQGMALLTSVHILADTYDN------------------------EMIFRAFVRDLMNRHKERG-------------------L----------DPKLW
OOP00190_1 : DRFAVQGMALLTSVHILADTYDN------------------------EMIFRAFVRDLMNRHKERG-------------------L----------DPKLW
OOP03513_1 : ERFAKQGTALVMSVHIFANLYDN------------------------DMVFRGFCRDLMNRHVERK-------------------L----------DPALW
OOP03348_2 : DRFATQGNALLTSVYILADTYDN------------------------EMIFRAFVRDLMNRHKERG-------------------L----------DPKLW
OOP03092_2 : ERFAKQGTALVMSVHIFANLYDN------------------------DMVFRGFCRDLMNRHVERK-------------------L----------DPALW
OOP00214_1 : DRFAKQGTALLMTVHIFANTYDN------------------------EMVFRSLCRDLMDRHVGRN-------------------V----------DPALW
PTP03438_1 : ERFEKQGQRLLLAVHILCETIDN------------------------PEVFKGYVREMVNRHRQFK-------------------L----------DPALW
OVP00634_1 : DRFAKQGQRVLLAVRILINTYDD------------------------SETFKAYARETVNRHIKFK-------------------M----------DRTLW
OVP04040_1 : DRFAKQGQRVLLAVRILINTYDD------------------------SETFKAYARETVNRHIKFK-------------------M----------DRTLW
SSP04654_1 : CGLREHIHFFVSLISQVIKALDTE-----------------------PQYIIEHINKIAIFHTNLKQYG----------------F----------RSLSF
SSP00231_1 : ERFAKQGVRIMLAMHILATAYDD------------------------QPTFLAYVRETINRHRIFK-------------------M----------PEELW
SSP02226_1 : ERFAKQGVRIMLAMHILATAYDD------------------------QPTFLAYVRETINRHRIFK-------------------M----------PEELW
Trichostrongylu : DRFKKLGNQLLLSVHLAADTYDN------------------------EMIFRAFVRDTIDRHVDRG-------------------L----------DPKLW
SSP04285_1 : KIFQNHAMTFTRSLDFCISNMDN------------------------LDVLCSYCYNLGRRHVAFAKRG----------------F----------KMSYW
Toxocara : EHFKKQGQTLLLACHVLAHLEND------------------------PSSFNAYCREIIDRHLRANVH-----------------L----------DPKLW
TCP00537_1 : EFFAKQGQKILLACHVLCATVDD------------------------METFNAYSHEVLDRHE-RDHVE----------------L----------PASAW
TDP00173_1 : ERFEKQGTALLMSVHIFANLYDN------------------------EMVFRAFCRDLIDRHVGRG-------------------L----------DPTLW
TDP00009_2 : ERFAKQGTALVMTVHIFANLYDN------------------------DMVFRGFCRDLMNRHVERK-------------------I----------DPALW
TDP00009_1 : ERFAKQGTALVMSVHIFANLYDN------------------------DMVFRGFCRDLMNRHVERK-------------------I----------DPALW
TDP00127_1 : DRFAKQGVALVMSVHIFANLYDN------------------------DMVFRGFCRDLMNRHVERK-------------------L----------DPALW
TDP00084_1 : DRFEKLGTGLLLSVHLLANTFDN------------------------EMVFRAFCRETIDRHVGRD-------------------L----------DPSLW
TDP00032_1 : DRFAKQGTALLMTVHIFANTYDN------------------------DMVFRSLCRDLMDRHVGRN-------------------V----------DPSLW
TDP00008_1 : DRFAVQGMALLTSVHILADTYDN------------------------EMIFRAFVRDLMNRHKERG-------------------L----------DPKLW
TDP00008_4 : DRFAVQGMALLTSVHILADTYDN------------------------EMIFRAFVRDLMNRHKERG-------------------L----------DPKLW
TDP00008_2 : DRFAVQGMALLTSVHILADTYDN------------------------EMIFRAFVRDLMNRHKERG-------------------L----------DPKLW
TDP00008_3 : DRFAVQGMALLTSVHILADTYDN------------------------EMIFRAFVRDLMNRHKER-G------------------L----------DPKLW
TDP01113_2 : DRFATQGSALLTSVHILADTYDN------------------------EMIFRAFVRDLMNRHKERG-------------------L----------DPKLW
TDP01113_1 : DRFATQGSALLTSVHILADTYDN------------------------EMIFRAFVRDLMNRHMERG-------------------L----------DPKLW
TMP01615_1 : VKFQKLGEDFLKQLNKFVDLADN------------------------EEALKNEVKQFMMNHKNYN-------------------V----------GAKEL
TVP00688_1 : QRFKTLGKLFLEKFKRIVMACED------------------------EEKLKEELKGLKLDHDPRH-------------------V----------GLTEF
TMP00180_1 : QRFKTLGKLFLEKFKRIVMACED------------------------EEKLKEELKGLKLDHDPRH-------------------V----------GLTEL
TVP00077_1 : AKFQQLGQDFLKQMNKFVDLASN------------------------EEALKMEVKQFMLNHKNYN-------------------V----------GAKEL
XIP00721_1 : ERFQKQGQRLLTSVHVLVEIWSN------------------------PPIYDAFVKDLMDKHKRFKG------------------V----------GYPHY
ZPP00218_1 : DRFRKQGQRILLACHILANTYDD------------------------EMTFKAYARETVNRHRQFK-------------------M----------DPPLW
MIP00306_1 : GRSQRYIMMAMNEIQALMQLPEQVK----------------------DERSWRSSLSNVKEHYSDSD------------------V----------PLSNF
RSP00034_1 : PKSARYIMMANNELQAFMQLPKNFN----------------------EERDWRSALSNFKEHYSDSD------------------V----------PLKLF
OVP06929_1 : EERIELANALRNYLKNVVNQLADG------------------------ANVQRISEDFGARHVQYRTFG----------------F----------RPDFF
GRP00115_1 : GRSQRYVMLAINELQAFVQMPTNL----------------------ADDRSWRSALSNFKEHYSDAD------------------V----------PLKYF
MAP00909_1 : CGVMEHTRFFLSLIDRIIEGDN--------------------------EGIECELRLIGARHVPCYGYFN---------------L----------NVTQL
HGP00385_1 : VPLEKQGQRILMAKQWIAKVDS------------------------NGMFSSAQGRETANSHCQ---------------------L--------KREPSLW
 H

helices : ggGggGGGgGGggg--------------------------------------------hhhhhhHHhhHHhhHhhhHhhhhhhh-----
residus : --|--|||-||-----------------------------------------------------||--||--|---|------------
ZK637.13 : MAFFTVFTGYLESVGC-----------------------------------------LNDQQKAAWMALGKEFNAESQTHLKNSNLPHV
CBG0687 : MAFFTVFTGYLESTGS-----------------------------------------LNDQQKAAWMALGKEFNAECQVHLKNSNLPYV
CRZK63 : MAFWTVFTGYLGSTGS-----------------------------------------LTDQQKAAWMELGKEFNAECQEHLKNSNLPYV
F49E2.4 : LVFKKVTVDQVTTGTTDSS--KEKE-D-TNSNGTANGKVDTDASLIPIADINNVYSGENCLARLGWNKLMTVIVREMKRGFLEEAMRNC
CBG16082 : LVFKKVTVDQVTTGATDSS--KEKDKDETNSNGTANGKVDTEANPIPIADINNVYSGENCLARLGWNKLMTVIVREMKRGFLEEAMRNC
CRF49 : LVFKKVTVDQVTTGATDSS--KEK--DETNSNGTANGKVDTDTNLIPIADANNVYSGENCLARLGWNKLMTVIVREMKRGFLEEAMRNC
C52A11.2 : NVFIERLIPFLTGEQE-----------------------LPSSEGKENKKVRFAQNYTTSQITDVWKKFLNTVISQMTDSFELERAKQK
CBG03023 : NVFIERLIPYLTGEQE-----------------------LPPAEGKEQKKVRFAQNYTTSQIADVWKKFLNIVISQMTDSFELERAKQK
CRC52 : NVFIERLIPFLTGEQE-----------------------LPPAEGKEHKKVRFAQNYTTSQIADVWKKFLNIVISQMTDAFELERAKQK
C28F5.2 : ERLGEIAMERVCSHEV---------------------------------------VTKTREASRAWRTLIAILIDELRGGFEGERQHRK
CBG13047 : ERLGEIAMERVCSHEV---------------------------------------VTKTREASRAWRTLIAILIDELRGGFEGELRQHR
CRC28 : ERLGEIAMERVCSHEV---------------------------------------VTKTREASRAWRTLIAILIDELRGGFEGELRQHR
W01C9.5 : DRFQEIMVEVILKQDG---------------------------------------VKQSKETSRAWRLLICSFIELIRDGFDAQVRQFR
CBG00571 : DRFQEIMVEVILKQDG---------------------------------------VKQSKEATRAWRLLICSFIELIRDGFDAQVRQFR
CRW01 : DRFQEIMVEVILKQDG---------------------------------------VKQSKETSRAWRLLICSFIELIRDGFDAQVRQFR
C29F5.7 : DIFLVCMAEKIDETLS----------------------------------AYIPDEDKRNEMILAWQRVINSIVHQMRNGYSDRRKQQL
CBG02622 : DIFLVCMAEKIDETLS----------------------------------SYMIEEDKKNEMILAWQRVVNCIVHQMRNGYSDRRKQQL
CRC29 : DIFLVCMAEKIDETLS----------------------------------SYMTEEDKKNEMILAWQRVVNSIVHQMRNGYSDRRKQQL
F19H6.2 : ETFAECMTQAAVEWEA----------------------------------------NRQRPTLGAWRNLISCIISFMRRGFDEENGKKK
CBG00138 : ETFAECMTQAAVEWEA----------------------------------------NRQRPTLGAWRNLISCIISFMRRGFDEENGKKK
CRF19H6.2 : ETFAECMTQAAVEWEA----------------------------------------NRQRPTLGAWRNLISCIISFMRRGFDEENGKKK
R01E6.6 : ADAMTLEGVILDMANQ-----------------------------------------HPADTVSAWSSLVTMIFSSVRDGYYSELRRHR
CBG07422 : ADAMTLEGVILDMANH-----------------------------------------QPADTVSAWSSLVTLIFSSVRDGYYSELRRHR
CRR01 : ADAMTLEGVILDMANH-----------------------------------------QPADTVSAWSSLVTLIFSSVRDGYYSELRRHR
F46C8.7 : EKFGEVMIDVVLAQEA---------------------------------------VRDLPGAGQAWVIFTACLVDQMRAGFDENRKTDH
CBG16720 : EKFGEVMIDVVLAQEA---------------------------------------VRDLPGAGQAWVIFTACLVDQMRAGFDENRKTDH
CRF46 : EKFGEVMIDVVLAQEA---------------------------------------VRDLPGAGQAWVIFTACLVDQMRAGFDENRKTDH
R13A1.8 : RVFCAQIVCTVFDFLR--------------------------------------DTEATPKCAESWIELMRYLGQKLLDGFDFAKLTAE
CBG05809 : RIFCAQIVCTVFDFLC--------------------------------------ETEATPKCAESWIELMRYLGQKLLDGFDFAKLTAE
CRR13 : RIFCAQIVCTVFDFLR--------------------------------------ETEATPKCAESWIELMRYLGQKLLDGFDFAKLTAE
C06H2.5 : DQLGEAFTELITKVEC---------------------------------------VRSKREAVKSWMCVISYMADSIKSGYMEEWAKKR
CBG23115 : DQLGEAFTDQKVECVR-----------------------------------------SKREAVKSWMCVISYMADSIKSGYMEEWAKKR
CRC06H2.5 : DQLGEAFTELITKVEC---------------------------------------VRSKREAVKSWMCVISYMADSIKSGYMEEWAKKR
C26C6.7 : NTVAETIIDCTLEWGD--------------------------------------RRCRSETVRKAWALIVAFVIEKIKAGHHEQRKLML
CBG11881 : NTVAETIIDCTLEWGD------------------------------------------RRTVRKAWALIVAFVIEKIKAGHHEQRKLMG
CRC26C6.7 : NTVAETIIDCTLEWGD--------------------------------------RRCRSETVRKAWALIVAFVIEKIKAGHHEQRKLML
F52A8.4 : DIFQDAMEFALEHRLS---------------------------------IMTDLDDNQKRDAVTVWRTLALYTTVHMRNGFIDGGLKGV
CBG11915 : DIFQDAMEFALEHRLS---------------------------------IMTDLDDNQKKDAVTVWRTLALYTTVHMRNGFIDGGLKGV
CRF52 : DIFQDAMEFALEHRLS---------------------------------TMADLDEAQKKDAVTVWRTLALYTTVHMRNGFIDGLKGVN
T22C1.2 : DKLGEAITETIREYQG-----------------------------------WKIHRESLRAATVLVSYVVDQLRFGYSRGLHVQGSRDT
CBG08252 : DKLGEAITETIREYQG-----------------------------------WKIHRESLRAATVLVSYVVDQLRFGYSRGLHVQGSRDT
CRT22 : DKLGEAITETIREYQG-----------------------------------WKIHRESLRAATVLVSYVVDQLRFGYSRGLHVQGSRET
C18C4.1 : QDSLLESMKLVLNGGD----------------------------------------SLDDDIEQSWICTSLRIPPGSFEYDNSK-----
CBG09371 : QDCLLESMRLVLNGGD----------------------------------------ALDDEIEHSWICLLQTIRLHMGEGIEIQRANYL
CRC18C4.1 : QECLLESMRLVLNGGD----------------------------------------SLDDEIEQSWICLLQTIRLHMGEGVEIQRANYL
R11H6.3 : EKVGEYFVDHVVIQDC---------------------------------------VRGFPEACRAWTVLVSSIVDRLRAAPRRGSFLNS
CBG04577 : EKVGEYFVDHVVIQDC---------------------------------------VRGFPDACRAWTVLVSSIVDRLRAAPRRGSFLNS
CRR11 : EKVGEYFVDHVVIQDC---------------------------------------VRGFPDACRAWTVLVSSIVDRLRAAPRRGSFLNS
C23H5.2 : GVFIECTLFHFRKVLG--------------------------------QDTYFHRMDALDKVIINWRIIIRLLIKQMKRGFNTDIKNRQ
CBG10551 : GVFIECTLFHFRKVLS--------------------------------QDSYFHRMETLDKVIINWRTILRLLIKQMKRGFNTDIKNRQ
CRC23 : GVFIECTLFHFRKVLG--------------------------------QDSHFHRMETLDKVIINWRTILRLLIKQMKRGFNTDIKNRQ
CBG24799 : QNMEEPFILMVKHVLQ---------------------------------------DRFNEKAEMLYRKFFQFCLKYLLEGFNG------
CRT06 : QNMEEPFIQMVKYILS---------------------------------------DRFNEKAEMLYRKFFQFCLKYLLEGFNG------
T06A1.3 : QNMEEPFIQMVSHILQ---------------------------------------DRFNEKAEMLYRKFFQFCLKYLLEGFNG------
Y17G7B.6 : EQFGEISVFVIGSQDV---------------------------------------VKFNHETVKAWRLLLACVTDEMKVGFDRMSRING
CBG21021 : EQFGEISVFIIGSQDV---------------------------------------VKFNHETVKAWRLLLACVTDEMKVGYDRMTRING
CRY17 : EQFGEISVFIIGSQDV---------------------------------------VKFNHETVKAWRLLLACVTDEMKVGFDRMTRING
Y57G7A.9 : EEFTLTMMGVLEQNYP----------------------------------------ETTKEEQKAWLHFLRFVNENMLDGYLDAISRSN
CBG07112 : EEFTLTMMDVLEQNYP----------------------------------------QTTKEEQKAWLHFQRFINENMLDGYLDALSYNN
CRY57 : EEFTLTMMDVLEQNYP----------------------------------------QTTKEEQKAWLHFQRFVNENMLDGYLDALSYNN
Y15E3A.2 : EKFGESLLTTATEWNG--------------------------------------PGRRHKETVKAWMVMSSFLADRLAHASRLAHHSPM
CBG07681 : EKFAEALMSVASEYNG--------------------------------------PGRRHRDIGRAWMLLSSFLADRLAHASRTSHQSPM
CRY15 : ENFAESLLNTATEWNG--------------------------------------PGRRHKETVRAWMLMTSFLADRLAHASRATAHASP
Y58A7A.6 : QTMYWDIFTDCFE---------------------------------------------RNPPPSFKKGAEREVALKFHRF---------
CBG08670 : QSMYWDIFTDCFE---------------------------------------------RNPPPSFKKGAEREV----------------
CRY58 : QSMYWDIFTDCFE---------------------------------------------RNPPPSFKKGAEREVRIQVISFRTSAEQPSE
C18C4.9 : DQFATAMIERTLEWGE--------------------------------------KKDRTETTQRGWTKIVLFVTEQLKEGFQDEQKRAR
CBG09371 : DQFATAMIERTLEWGE--------------------------------------KKDRTETTQRGWTKIVLFVTEQLKEGFQDEQKRAR
CRC18C4.9 : DQFATAMIERTLEWGE--------------------------------------KKDRTETTQRGWTKIVLFVTEQLKEGFQDEQKRAR
R90.5 : DDLGDTIMDCARRRCE--------------------------------------AVRKHKELRRAWLAIIAYIMDNLKQGQSMTRSSST
CBG09511 : DDLGDTIMDCARRRCE--------------------------------------AVRKHKELRRAWLAIIAYIMDNLKQGQSVTRASSS
CRR90.5 : DDLGDTIMDCARRRCE--------------------------------------AVRKHKELRRAWLAIIAYIMDNLKQGQSMTRSSSS
C36E8.2 : CTADAVTTECTFLDQA---------------------------------------AHPTSETAAAWSLLTSHVFSAVRDGYYAELRRQR
CBG03635 : CTADAVTTECTFLDQA---------------------------------------AHPTSETAAAWSLLTSHVFSAVRDGYYAELRRQR
CRC36 : ATADAVATECTFLDQA---------------------------------------AHLTSETATAWYLLTTHVFSAVRDGYYAELRRQR
Y75B7AL.1 : KEFKHTIQTIISEVQF-------------------------------------SSPQEREAALDAWNIFISFIIREMKMGIWAIGDTIG
CBG06424 : KEFKYTIQSIISEVQF-------------------------------------SSPQEREAALDAWNIFISFIIREMKMGIWAIGDTIV
CRY75 : KEFKNTIQTIISEVQF-------------------------------------TSPQEREAALEAWNIFISFIIREMKMGVWAIDDAIG
F35B12.8 : LIFKNVLLDLLCKDA-------------------------------------------NEKVGATWNKLISFMISEVKDSYLEHVRHAR
CBG23302 : LIFKNVLLDLLCKDA-------------------------------------------NEKVCGTWNKLISFMISEIKDSYLEHVRHAR
CRF35B12.8 : LIFKNVLLDLLCKDA-------------------------------------------NEKVCGTWNKLISFMISEIKDSYLEHVRHAR
F21A3.6 : IHMIEPVLEVVKECNG---------------------------------------YQLDDETRQAWTVLYQVIADLIEVFRCRALND--
CBG18593 : IHMIEPVLEVVKECNG---------------------------------------YQLDDETRQAWTVLYQVIANLIEVFRCRALND--
CRF21A3.6 : IHMIEPVLEVVKECNG---------------------------------------YQLDEETRQAWTVLYQVIANLIEVFRCRALNE--
C06E4.7 : LCVKNCLLDSVVSALL-----------------------KDTKGTYVICGGLKKVQTVEKHTTHAWFKFVQFIIQNMKKGFLAEALNSD
CBG05824 : LCVKNCLLDSVVSALL-----------------------KDTKGTYLICGGIKKVQTVEKHITHAWFKFVQFIIQNMKKGFLSEALNSD
CRC06E4.7 : LCVKNCLLDSVVSALL-----------------------KDVKGTYVICGGLKKVQTVEKHTTHAWFKFVQFIIQNMKKGFLAEALNSD
F56C4.3 : SVFLECSIYCLRHAFS-----------------------------------KRMNDKEVDHVIILWRYLLRDVMKKIKAGTTADIAHRL
CBG04428 : SVFLECSIYCLRHAFS-----------------------------------KRMNDKEVDHVIILWRYLLRDVMKKIKAGTTADIAHRM
CRF56 : SVFLECSIYCLRHAFS-----------------------------------KRMNDKEVDHVIILWRYLLRDVMKKIKAGTTADIAHRM
R102.9 : RGVNFGADNWLTFKKV------------TVEIVTKDCGNSESSSMDLKSVPSLFPSSSSTVIIIGWEKFMSSVIREMKKGFLDEARRNC
CBG17640 : RGVNFGADNWLTFKKV------------TVESVTQDGGSSESSVIDMKSVPSLFPSNSSTVVIIGWEKFMSSVIREMKRGFLDEARRNC
CRR102 : RGVNFGADNWLTFKKV------------TVESVTQDGGSSESSVIDMKSVPSLFPSNSSTVVIIGWEKFMSSVIREMKRGFLDEARRNC
C09H10.8 : QQLPIAMSKMYMRVVT----------------------EDSKIRRILRIRSRAAEKSEEEEVCESWRQFSCMLIESMKRGYEGCASEKT
CBG02965 : QQLPIAMSKMYMRVVN----------------------EDSKIRRILRIRSRAAEKSEEEEVCESWRQFSCMLIESMKRGYEGCASEKT
CRC09 : QQLPIAMSKMYMRVVT----------------------EDSKIRRILRIRSRAAEKSEEEEVCESWRQFSCMLIESMKRGYEGCASEKT
Y22D7AR.5 : SVFKECVLFHFRKAMK---------------------------SHNKFHKRNEMSFAEIDSAIILWREVLRFIIDRMKVGYSESGAIRK
CB_Y22 : SVFKECVLFHFRKAMK---------------------------SHNKFTKHKEMSFAEIDSAIILWREVLRFIIDRMKVGYCES-----
CR_Y22 : SVFKECVLFHFRKAMK---------------------------AHNKFSKHKEMSFAEIDSAIILWREVLRFIIDRMKVGYCES-----
Pp27.56 : ILFEDAMITAMKKVLKGVE-------------------------------------ELDEETERSWRVLLQLTRKHLIEGISIQR----
Pp42.55 : DDLADSLMECVCRY-DAV--------------------------------------KKHKELRRAWLALIAYIVDNLKNLRNRRQR---
Pp10.104 : NLVAETFIDCTLEWGDKR--------------------------------------CRSETVRKAWALIIAFMVEKIKLGHHEQR----
Pp94.7 : KEFKACLLGIIGELDYDSP-------------------------------------KAMCTPRSPSHATKN------------------
Pp50.31 : SVFKECTLYHFRKAMRGDKRNRPLSRYCIRSSHKAKMTP-----------------SEIDSAIILWREVLRVMIERMNAGLEENN----
Pp106.6 : DVFTDCFENNPPATF------------------------------------------KTDIDREAWSAMILFILAQMKLGFRQVD----
Pp68.42 : DVFLVCMGEVLSEVLLSYTTDA----------------------------------GRRAEISMACERVFATTVHYMRTGYQERRRKEA
Pp14.29 : LVFKRVTVDQVTKGVTSTQASQANLLEGTKEPEVVEQHPMADVQNPFSG--------ENCLARLGWNKLMTVIVREMKRGFLEEA----
Pp84.53 : EKLGETIIDVVLAQEAVRD---------------------------------------LPGAGQAWVVLTACLVDQV------------
Pp68.66 : DIFMACLRDAFQESMATFGTER----------------------------------DEELEIL--FEKTFAWVLYNMRLGFQDRK----
Pp168.42 : DVFLDAMQYAQDQRIPKMNNLNA---------------------------------QEKQRAKQVEEGKGDTV----------------
Pp326.1 : NVFKDSVQAGIKNRLNSLPDLSA---------------------------------QERSRAVVIWRDIIEYIFEYVKEGFYDGL----
Pp2.166 : DVFKDAVRTGLNDRLNSLAKLSA--------------------------------EERIRAIEVIWADIIEYIFEHVKEGFYDGL----
Pp2.164 : VAFQDAMQNVITEKMKSITKISE---------------------------------TDRARAIEIWKDVVVYVNTNMKAGYEDGL----
Pp82.11 : AIFKDAIQAGLNDRVNRLHELSQ---------------------------------EERFRAVSLWRDIIEYMFVYVKEGFYDGL----
Pp360.2 : AGTADAVTTECTFLDGAT-------------------------------------HAPSETAGAWYSTPSFHRVVLFSDGYYQEL----
Pp14.30 : LAFFTVFVGYLGTK-----------------------------------------TTLDEATKNAWAEMGKVRCAKIVCAYSSLN----
Pp38.62 : ETFAECMTQSAAEWEQH----------------------------------------RHRPTTSAWRSLSKGCGSMWNSVKKRDS----
Pp5.100 : DSFAECMTEAAAEWELH----------------------------------------RNRPATSAWRILVSNIIMFMRRGFDDEI----
Pp277_1 : EIVLNVFEQRLIPYLTG-------------------EKEEPVPEGQAPRKVRFAQSYTPDVVTAVWKKFFAILVTQMTDAFELER----
Pp43.86 : ERLGEIVMERICGLESCQ---------------------------------------KTREASRAWRTLIACVIDELRTGFEEEH----
Pp94.50 : ENLGEMLTQVITRNDCVR---------------------------------------QKREAVKAWIGLISYL----------------
Pp137.9 : ESMLPELFDVLKECMGG---------------------------------------TVTRETVYAWTTLYDIIGPNSKLYIS-------
Pp38.51 : VALADAITVEGVILDMAN--------------------------------------HQPADTVAAWSQLVTLMFSSVRDGYYTAL----
Bm1_37235 : SVFLECSIHHIRLALLSSK-------------------------------ADRWNNTDVDNVVILWRHLISGICERIKRGYLTNIADRS
Bm1_31825 : VSIADAMAVECVILDMAN--------------------------------------HQPTETVMAWSQLTSLMFTSIRDGYYAAL----
Bm1_41355 : QKMIKPLLENVRRALG----------------------------------------RHDENAERAWETLFQTVGAIVEHYKTSQV----
Bm1_13755 : XITADAVTTECVLLDAAVHS--------------------------------------ASEALFAWSTLTTFMFSSVRDGYYSEQ----
Bm1_27145 : SVLADALTTECVFLDGAAHQ--------------------------------------PTETIEAWATLVELMFTNVRDGYYMET----
Bm1_17795 : DQFGDSLAESIAKAEAI---------------------------------------RGKRKCLKAWNALLSFIVDRIKGGYLAES----
Bm1_02240 : NLMAEAFIDCTLDWGDK--------------------------------------KGRTEASRKAWAFIISFAIEKIKRGHLHER----
Bm1_33065 : EKLGEIIVDVVLVQEAVR---------------------------------------DLPGAGQAWVIFIACLVDQLRAGFDESK----
Bm1_21435 : DTFNDCMRQTVSLWGKD----------------------------------------KDHRTANTWHTLISFVLQNMKIGFNRAN----
Bm1_50430 : LAFFTVLVNSLKEH-----------------------------------------TIIDEEAEKAFLQIGKEFSDECLKHIIALN----
Bm1_46940 : AVVGNTFIDEFIPYLNG----------------------EKEEPSQDKKRVRFASTYSVTMISDVWRRFFTILVAQITESFEQER----
Bm1_24705 : EKLGEITMQCFSRQDAVQ---------------------------------------KTREAGKAWRILIAWVTDELRCGFDGRT----
Bm1_04635 : RLFCSQVVCAVADLLEVD---------------------------------------IDPACMEAWIDMMRFIGCRLLDGFNYIR----
Gossypium : EVTKFALLETIKEAVP---------------------------------------DMWSDEMKNAWGEAYDRLVAAIKIEMKACSLAA-
Malus : EVTKFALLETIKEALP---------------------------------------EMWSPEMKNAWGEAYDQLVAAIKSEMKPPLN---
Glycine : EVTKFALLETIKEAVP---------------------------------------EMWSPAMKNAWGEAYDQLVDAIKSEMKPPSS---
Alnus : EVTKFALLETIKEAVP---------------------------------------EMWSPEMKIAWGEAYDQLVAAIKSAMKPSS----
Citrus : EVTKYALLEPIKEPVP---------------------------------------EMWSPELKNAWAEAYDQLAAAIKIEMKPPS----
Nemvec141000032 : EAMHGALMDTLRNLLQS---------------------------------------QWTEETAEAWNKLFSFISTTMVRGLQSRD----
Nemvec3000224 : GVVGEAFIWALQDVLGE---------------------------------------GCTSDVAEAWIDLYGYIVQAMLEGLQQAKKGR-
Nemvec50000067 : KFMGGAFLFAMRLHLRK---------------------------------------EWSRATSKAWEKIFSFMADAMMRGCKG------
Nemvec7000121 : EPVGKALIYSISKGLGS---------------------------------------LFNDEIGEAWQAMYDLMSGAMISGTKAVQARSQ
Nemvec76000030 : QEISQAINETFEENLGI---------------------------------------KWTVEIAESWKLLLDYVMAMIIRGLRSP-----
Paragonimus1 : LSGEPIFIDFFNKTLS------------------------------------------KPENKAAMEKFLKHAFPVIANNI--------
Paragonimus2 : LSGEPVFIKYFNELLT------------------------------------------KDENKAAMEKLLKHVMPAIASKI--------
Clonorchis : LSAEEVFIKHFSGVLTKE--------------------------------------ENKQSMERFLKHIVPKVAGFLG-----------
Isoparorchis : QTGEPIFIKYFSHVLTTP--------------------------------------ANQAFMEKLLTKIFTGVAGQL------------
Paramphistomum : MSGEPIFTKYFQNLVK------------------------------------------DAEGKAAVEKFLKHVFPMMAAEI--------
Schistosoma : QDSLPIFIKIFNNLIKD------------------------------------------QQNKETMSKILTYTFTMIGSQI--------
Pseadoterranova : HEFWKLFAEYLNEKSHQ---------------------------------------HLTEAEKHAWSTIGEDFAHEADKHAKAE-----
Heterorhabditis : GAFFEVFKGFLDSRGGI-----------------------------------------TDTQKTAWDQLEKFSTMSASSISNIS-----
Mermis_Eye : SAFFDVWYGYLSKII-----------------------------------------GMSDKEKKEWEAFRVELFLPAVKKYIAG-----
Mermis_Body : NAFFDIWYGYLSKVI-----------------------------------------GLSDQEKKEWEAFKVELFLPAVKKYIGG-----
Toxoperient : TDFWKIFIEYLEKKMTV-----------------------------------------DEATKNAWMEIGKDFATAVGQHESSH-----
GRP00348_1 : VSLADAMTVECVILDQAT--------------------------------------HQPSDTITAWSLLVSIMFSAVRDGYYQA-----
ACP03829_1 : KAFWPIWMAFLESK--GA--------------------------------------TLSADQKAAWDALGTTFNDECQQQLAKH-----
ACP01487_2 : SAFFTVYVNFLASRGP-----------------------------------------LSDEQKKAWAQLGKVFDEECQSHLKEL-----
ACP00369_1 : STFLECSICIFRKTLANCRKYPD---------------------------------KDIDHAIILWRYLLRDVMKKIKAGYNAD-----
ALP00043_1 : SEFWPIWLDYLSTKES-----------------------------------------VDDATKNAWLALGKKFSDECLDHLKNL-----
AscarisMb : SEFWPIWLDYLSTKES-----------------------------------------VDDATKNAWLALGKKFSDECLDHLKNL-----
AscarisHb : TDFWKLFEEYLGKKT-----------------------------------------TLDEPTKQAWHEIGREFAKEINKHGRHA-----
Syng_Intracell : SAFFTVFVNFLETRGS-----------------------------------------LTDDQKKAWAQLCKVFDEECQSHLKTL-----
Syng_Cuticul : KDFWKIWTAFLESKGT----------------------------------------TLSADDKAAWEALRDRFNDESQKELAKR-----
ASP00019_1 : TDFWKLFEEYLGKKT-----------------------------------------TLDEPTKQAWHEIGREFAKEINKHGRHA-----
ASP00780_1 : SAFFTVFTEYLATKG-----------------------------------------ADDDATKKAWQEVGKEFSTECLAHLKNL-----
ASP17423_1 : TAFWPIWLDYLATKT-----------------------------------------TVDDATKNAWLALGKKFADECCNHLKNL-----
AYP02881_1 : ERLGEITMERICAHEL----------------------------------------VQTREAARAWRVLLACIIDELRGGFDGE-----
AYP03578_1 : KAFWDIWMAFLESK--GA--------------------------------------SLSADQKAAWNALGTTFNEECQSHLATL-----
AYP00272_1 : KAFWPIWMAFLESK--GA--------------------------------------TLSADQKAAWDALGTTFNEECQQQLAKH-----
AYP01816_1 : KEFWGYWKAFLESK--GT--------------------------------------SLSAEQKEAWDTLGNLFNEEAQVQLAKH-----
AYP00544_1 : KEFWGYWKGFLENK--GT--------------------------------------TLSAQQKEAWDTLGKLFNEEAQSQLTKH-----
DIP00455_1 : DTFHDCMRQTVSLW----------------------------------------GRDKDLKTANTWHMLIS------------------
HCP00759_3 : KAFWGIWIAFLESK--GA--------------------------------------SLSGDQKAAWEKLGTTFNEECQSHLAKL-----
HCP00759_2 : KAFWGIWIAFLESK--GA--------------------------------------SLSGDQKAAWEKLGSTFNEECQSHLAKL-----
HCP00759_1 : KGFWPIWIAFLESK--GA--------------------------------------SLSGDQKAAWEKLGTTFNEECQSHLAKL-----
HCP00786_1 : KGFWGIWTAFLESK--GA--------------------------------------SLSGDQKAAWEKLGATFNEECQQHLARL-----
HCP13111_1 : KAIWSSIESFLETR--GT--------------------------------------SLTAEQKAALEAIGNKFNEEAQKDLAAH-----
HCP00770_1 : KDFWGIFEKFLESRKP-----------------------------------------LTADQKTALDTMGTRFNDEAQKQLAVL-----
HCP00770_3 : KDFWGIFEKFLESRKP-----------------------------------------LTADQKTALDTMGTRFNDEAQKQLAVL-----
HCP00208_1 : KEIWSSIESFLETR--GT--------------------------------------SLTAEQKAALEAIGNKFNEEAQKDLAAH-----
HCP00202_4 : KDFFGCFEKFLEGR--GK--------------------------------------PLTDDQKTALEAVGTKFNEEAQKHLAAL-----
HCP00208_2 : KAIWSSIESFLETR--GT--------------------------------------SLTAEQKAALEAIGNKFNEEAQKDLAAH-----
HCP01314_3 : KDFWTCFEDFLEGR--GK--------------------------------------PLTAEQKAAFEAIATKFNEEAQKHLASL-----
HCP00202_3 : KDFFGCFEKFLEGR--GK--------------------------------------PLTDDQKAALEAIGTKFNEEAQKHLASL-----
HCP00333_2 : KDFFGCFEKFLEGR--GK--------------------------------------PLTDDQKAALEAIGTKFNEEAQKHLATL-----
HCP00202_2 : KDFWTCFEDFLEGR--GK--------------------------------------PLTAEQKAAFEAIATKFNEEAQKHLASL-----
HCP00202_1 : KDFFGCFEKFLEGR--GK--------------------------------------PLTDDQKAALEAIGTKFNEEAQKHLAAL-----
HCP00770_2 : KDFWGIFEKFLESRKP-----------------------------------------LTADQKTALDTMGTRFNDEAQKQLAVL-----
HCP00202_5 : KDFFGCFEKFLEGR--GK--------------------------------------PLTDDQKAALEAIGTKFNEEAQKHLATL-----
HCP02815_1 : KDFWAIFEKFLNER-----------------------------------------KALTADQKTAIETLGTRFNEEAQKQLGVL-----
HCP08501_1 : KAFWDIWVAFLDSH--GT--------------------------------------VLSAEQKTAWNKLGTRFNEECQQYLAKQ-----
HGP06223_2 : KAFWTVWTGFLEAKVD-----------------------------------------LTEQAKSAWMSLGEDXAKEALAHLKRL-----
MJP04640_1 : MAFWTVWTGFLATKIS-----------------------------------------LDEGHKNAWMTLGKDFAKAANKHLKLL-----
MIP00586_2 : MAFWTVWTGFLATKIT-----------------------------------------LDERHKNAWMTLGQDFAKAANKHLKLL-----
MHP02564_2 : MAFWTVWTGFLANKVG-----------------------------------------LDERHKNAWMALGQDFAKAANKHLKLL-----
NAP00088_1 : KSFWGIWMGFLESKGAT----------------------------------------VSGDQKAAWETLGTMFNEECQSHLSKL-----
Nippo_Body : KAFWSVWVAFLESKGG-----------------------------------------VSGDQKAAWDKLGTVFNDECQHQLAKH-----
Nippo_cuticul : KIFFDDVWVPFLESKGA---------------------------------------KLSGDAKAAWKELNKNFNSEAQHQLEKL-----
NBP00095_1 : KAFWGVWVAFLESKGS-----------------------------------------VSADQKAAWDKLGTVFNNECQQQLAKH-----
NBP00124_1 : KAFWGIYTAFLESR--GT--------------------------------------ALSADQKAAWEQIGTMFNDEAQQQLAKH-----
NBP00197_1 : TIFFDDVWVPFLESKGA---------------------------------------KVSGDTKAAWKELNKNFNGEAQHQLEKL-----
NBP00328_1 : KIFFDDIWVPFLESKGA---------------------------------------KVSDDTKAAWKELNKKFNSEAQHQLEKL-----
OOP00190_2 : KDFWDIFEKFLENRKP-----------------------------------------LTADQKAALDAMGTRFNDEAQKQLAVL-----
OOP00190_1 : KDFWGIFEKFLENRKP-----------------------------------------LTADQKTALDAMGTRFNDEAQKQLAVL-----
OOP03513_1 : KAFWGIWTAFLESKGA----------------------------------------SLSGDQKAAWEKLGTTFNEECQQHLAKL-----
OOP03348_2 : KEFWGIFEKFLDGRKA-----------------------------------------LTADQKTAIETLGTRFNDEAQKQLAVL-----
OOP03092_2 : KSFWGIWTAFLESK--GA--------------------------------------SLSGDQKAAWEKLGTTFNEECQQHLAKL-----
OOP00214_1 : KGFWGIWIAFLESK--GA--------------------------------------TLSGDQKAAWEKLGATFNEECQQHLAKL-----
PTP03438_1 : LAFFTVWVAFLETKQT-----------------------------------------LSDDTKKAWQEMGKAFNDEAQRQLKSM-----
OVP00634_1 : AKFFTLFVNNLKEHTT-----------------------------------------VDEETEKAFQQIGKEFSDECIKHTVAL-----
OVP04040_1 : AKFFTLFVNNLKEHTT-----------------------------------------VDEETEKAFQQIGXKNFLMKCIKHTRA-----
SSP04654_1 : DRLGELLVDSLVVQETV---------------------------------------RCFPEACKAWTILIAAMTDKLRTPSSSL-----
SSP00231_1 : DAFFPVWLGFLQTKGV-----------------------------------------VTPEVTNAWNQIGKTFTSEAHRYIKSN-----
SSP02226_1 : DAFFPVWLGFLQTKGV-----------------------------------------VTPEVTNAWNQIGKTFTSEAHRYIKSN-----
Trichostrongylu : KEFWSIYQKFLESKGK----------------------------------------TLSADQKAAFDAIGTRFNDEAQKQLAHH-----
SSP04285_1 : DTFAEALTECAIKWEGG---------------------------------------FRCXDILQGWRKL--------------------
Toxocara : TAFWPIWLDYLATKT-----------------------------------------TVDDATKNAWLALGKKFADECCNHLKNS-----
TCP00537_1 : TDFWKIFIEYLEKKM-----------------------------------------TVDEATKNAWMEIGKDFATAVGQHESSH-----
TDP00173_1 : KAFWGIWVAFLESK--GA--------------------------------------TLTADQKAAWEKLGTLFNEECQLQLAKH-----
TDP00009_2 : KAFWGIWIAFLESK--GA--------------------------------------SLSGDQKAAWEKLGTTFNEECQSHLAKL-----
TDP00009_1 : KGFWPIWIAFLESK--GA--------------------------------------SLSGDQKAAWEKLGTTFNEECQSHLAKL-----
TDP00127_1 : KSFWGIWTAFLESK--GA--------------------------------------SLSGDQKAAWEKLGTTFNEECQSHLAKL-----
TDP00084_1 : KGFWGIFVAFLESR--GT--------------------------------------ALTADQKAAWEKLGTMFNEECQLQLAKH-----
TDP00032_1 : KGFWGIWTAFLESK--GA--------------------------------------SLSGDQKAAWEKLGATFNEECQQHLAKL-----
TDP00008_1 : KDFWGIFEKFLESRKP-----------------------------------------LTADQKTALDTMGTRFNDEAQKQLAVL-----
TDP00008_4 : KDFWGIFEKFLESRKP-----------------------------------------LTADQKTALDTMGTRFNDEAQKQLAVL-----
TDP00008_2 : KDFWGIFEKFLESRKP-----------------------------------------LTADQKTALDTMGTRFNDEAQKQLAVL-----
TDP00008_3 : KDFWGIFEKFLENRKP-----------------------------------------LTADQKTALDTMGTRFNDEAQKQLAVL-----
TDP01113_2 : KDFWGIFEKFLNER-----------------------------------------KALSADQKTAIETLGTRFNEEAQKQLGVL-----
TDP01113_1 : KDFWGIFEKFLNERK-----------------------------------------ALSADQKTAIETLGTRFNEEAQKQLGVL-----
TMP01615_1 : EKAEPAWMAFLESKTG-----------------------------------------VTPEQRAAWHAFFAKFMQLVTTF---------
TVP00688_1 : KGAKPILLKFMEHEVG-----------------------------------------MNEDQKKAWAEMFKKFETVYSSLHXTR-----
TMP00180_1 : KGAKPILLKFMEQQVG-----------------------------------------MNDEQKKAWAEMFKKFETVYSSLHYPL-----
TVP00077_1 : EKAEPAWMAFLESKTG-----------------------------------------VTPEQRGGLDGFFVKFMQLVSMF---------
XIP00721_1 : EAFFSVFRGYLEKLIGP----------------------------------------LSHEENKAWDAMKEDFLKTCKPYI--------
ZPP00218_1 : AAFFKVFVGYLEDKQHKA--------------------------------------PLDEKTKQAWEQLGDEFAKECWRHLKDQ-----
MIP00306_1 : IKTKDAWLAIMQKYAGG----------------------------------------LSAEQKKEWEELFTKASSDMKKWGWT------
RSP00034_1 : NKTKEPFLALLQKHAGG----------------------------------------MSAEQKKGWEELLDKAYADMKKYGXDL-----
OVP06929_1 : ATEPQMRTTECVLLDAR---------------------------------------YIASEALFAWGNIDSFHVQFGT-----------
GRP00115_1 : GKTKDAFLTVLQKHAG----------------------------------------GLNAEQKKNWEELMEKANADMKKWXLAL-----
MAP00909_1 : EQLGEALAGTFFKLEG----------------------------------------IKQAKNYKSLATLIAHIIDYIRE----------
HGP00385_1 : KALCTVRTGFSETKVD-----------------------------------------PAEQAKLASDVSGGGFC---------------
