# Supplementary figures and images for: The Caenorhabditis globin gene family reveals extensive nematode-specific radiation and diversification
Source: BMC Evol Biol. 2008 Oct 9;8:279. doi: 10.1186/1471-2148-8-279 (PMC2576238; doi:10.1186/1471-2148-8-279)

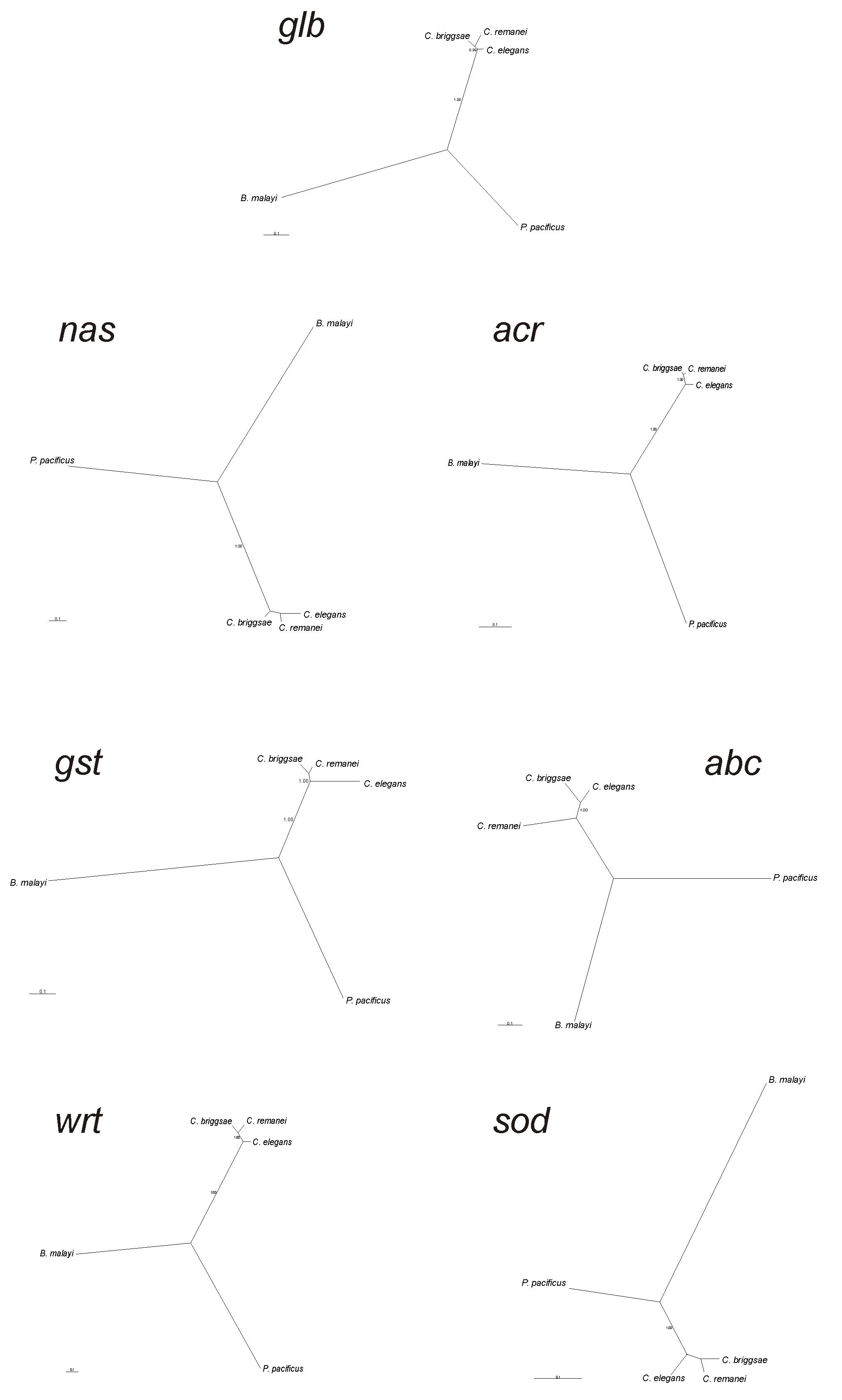

Supplement: Additional file 4 — Unrooted bayesian trees based on matrices of five-way orthologs from C. elegans, C. briggsae, C. remanei, Pristionchus pacificus and Brugia malayi. glb globin; gst glutathione S-transferase; ast astacin, sod superoxide dismutase, acr nicotinic acetylcholine receptors; wrt hedgehog-related (warthog) proteins; abc ATP-binding proteins. [file 1471-2148-8-279-S4.jpeg]
